# Substrate and low intensity fires influence bacterial communities in longleaf pine savanna

**DOI:** 10.1038/s41598-022-24896-x

**Published:** 2022-12-03

**Authors:** Viet Q. Dao, Stephen E. Potts, Crystal N. Johnson, Benjamin A. Sikes, William J. Platt

**Affiliations:** 1 Department of Environmental Sciences, Louisiana State University, 1273 Energy Coast and Environment Building, Baton Rouge, LA 70803 USA; 2 Department of Biological Sciences, Louisiana State University, Baton Rouge, LA USA; 3 Department of Biological Sciences, University of Kansas, Lawrence, KA USA

**Keywords:** Community ecology, Fire ecology, Microbial ecology, Community ecology, Fire ecology, Microbial ecology, Bacteria, Microbial communities

## Abstract

Bacterial communities associated with vegetation-soil interfaces have important roles in terrestrial ecosystems. These bacterial communities, studied almost exclusively in unburnt ecosystems or those affected by rare, high-intensity wildfires, have been understudied in fire-frequented grasslands and savannas. The composition of ground-level bacterial communities was explored in an old-growth pine savanna with a centuries-long management history of prescribed fires every 1–2 years. Using 16S metabarcoding, hypotheses were tested regarding differences in bacterial families of litter and soil surface substrates in patches of ground layer vegetation that were naturally burnt or unburnt during landscape-level prescribed fires. Litter/soil substrates and fire/no fire treatments explained 67.5% of bacterial community variation and differences, driven by relative abundance shifts of specific bacterial families. Fires did not strongly affect plant or soil variables, which were not linked to bacterial community differences. Litter/soil substrates and the naturally patchy frequent fires appear to generate microhabitat heterogeneity in this pine savanna, driving responses of bacterial families. Prescribed fire management may benefit from considering how fire-altered substrate heterogeneity influences and maintains microbial diversity and function, especially in these fiery ecosystems. Frequent, low-intensity fires appear ecologically important in maintaining the diverse microbial foundation that underlie ecosystem processes and services in fire-frequented habitats.

## Introduction

Savannas and grasslands are the most pyric ecosystems on Earth. Global distribution of these ecosystems, which comprise ~ 40% of the terrestrial biosphere^1^ is related to climatic and fuel conditions producing frequent ground-level fires^2^. Two of the multiple interacting processes that strongly influence frequent-fire dynamics, and are most prominent in mesic savannas and grasslands, interface in relation to fine fuels at ground level: plant production that forms an interconnected flammable matrix of live and dead fuels across the landscape^3^ and microbial decomposition of those fuels, in litter and adjacent soil^4^. Feedbacks involving accumulation and decomposition of those fuels result in biotically modified fire regimes^5^ that influence the composition and dynamics of these ecosystems^6^. Most concepts related to fine fuels in fire-frequented ecosystems, however, are based on production by plants^7^, ignoring decomposition by microorganisms. In ecosystems with less-frequent fire, plant production might readily exceed decomposition, but in ecosystems where fires are occurring almost every year, the production of fuels relative to decomposition of those fuels might have potentially large effects, especially with respect to fine fuel continuity across the landscape^8^.

Frequent fires in mesic savannas and grasslands affect ground-level microbial communities and fuel dynamics. Post-fire, fuel production by grasses is increased^9^, and decomposition of fine fuels is slowed^10^, enhancing short return periods of low-intensity fires that consume the bulk of fine fuels in each fire. Thus, litter is almost continually present, but tends not to accumulate. The frequent fires have been shown to reorganize fungal communities in both litter and soil, resulting in shifts in composition during the intervals between fires^11^. Post-fire responses of fungi affect fuel dynamics via decomposition^12^ and plant community recovery^13^; however, these effects are less studied in systems affected by frequent, lower intensity fires. Little is known, however, about responses of ground-level bacterial communities.

Studies of fire effects on microbial communities have largely focused on high fire intensities. Many studies have involved wildfires in non-fire-frequented systems^14^. In those systems, large fuel loads and wildfires coinciding with extreme weather events (e.g., drought) tend to produce severe effects. Increased fuel combustion and soil heating in these wildfires tend to cause extensive mortality and near sterilization of the upper soil layers. In contrast, fires in naturally pyric ecosystems are often less severe^15^, and these less intense, but frequent savanna and grassland fires should influence ground-level bacterial communities in different ways than high intensity wildfires. Lower intensity fires may have weaker effects than high intensity fires on bacterial community composition^16^, owing to more subtle effects on litter combustion and reduced heating despite less soil insulation^17^. Moreover, when fires occur frequently, variation in effects of individual fires should generate pyrodiversity that includes subtle mosaics of litter and soil properties^5^ that should affect post-fire bacterial communities^18^.

Studying bacterial community responses to frequent fires expands knowledge of fire ecology by identifying post-fire bacterial impacts on subsequent fires. Bacterial post-fire interactions may increase (e.g., via rhizobial symbioses) or decrease (e.g., via diseases) fuel production by plants. Further, bacteria, in tandem with fungi, decompose new fuels, so bacterial post-fire responses may contribute to observed short-term changes in fuel decomposition^10^, especially since bacteria are considered less sensitive than fungi to effects of individual fires and have faster growth rates than fungi^19^. Bacteria may play a larger role in post-fire environments since their resistance and resilience may maintain decomposition and symbiotic nutrient cycling functions post-fire in fire-frequented environments. Study of post-fire bacterial community responses should expand concepts that relate fire as an ecological process^20^ to ground-level fuel dynamics and fire feedbacks, as well as compositional variation in savanna/grassland microbial communities^21^. Understanding post-fire bacterial feedback effects on fuel decomposition and plant community recovery will help predict microbial effects on fuel dynamics and thus fire regimes, as well as microbial and functional adaptations to changes in fire regimes related to both climate and human effects^22–25^. This new element of microbial effects in fire ecology may also be useful in guiding management of ecosystems, especially those with old-growth characteristics that reflect long evolutionary histories in association with fire, especially in high diversity hotspots of biodiversity and endemism^22,23^.

Three hypotheses were explored, related to effects of frequent ground-level fires on litter and soil bacterial communities in savannas. First, frequent savanna and grassland fires produce a natural landscape mosaic comprised of variable-sized, but mostly small patches of unburnt vegetation and litter scattered within a broader landscape of burnt vegetation and variably combusted litter^10,26^. This mosaic shifts with each fire as patches burn that were not burnt in previous fires, but new unburnt patches are generated^26^, which describes the patchy quality of these frequent, low intensity fires. The differences in fuels, and thus fire intensities^27,28^ in burnt patches, coupled with unburnt patches, generate site-level pyrodiversity^5^ in each fire that were hypothesized to influence bacterial communities. Like fungal communities^10^, bacterial communities were anticipated to differ between burnt and unburnt patches. Second, overstory longleaf pine (*Pinus palustris*) trees that are scattered across the savanna landscape^27,28^ produce pyrogenic fuels that result in locally increased fire intensity^5,29^. Differences in bacterial communities after fire should be greater in close proximity to pines than away from pines due to increased fire intensities. Third, bacterial communities at ground level are associated with substrates (e.g., litter and soil) that differ in chemical composition and exposure to fire. Thus, interactions of substrate with fire should affect bacterial communities differently, such that the bacterial communities in litter, which are directly affected by fuel combustion, should shift more with fire than bacterial communities in the more insulated surface soil layer directly below litter. Given the role of local fire intensity, these differences should be magnified near compared to away from pines.

These hypotheses were explored related to effects of frequent fire on ground-level bacterial communities. This study was part of an interdisciplinary study of the responses of ground level plant and microbial communities to fire in an old-growth pine savanna. Litter and soil were sampled in patches of ground layer vegetation that were naturally burnt and unburnt, and located near and away from pines. Using 16S rDNA barcode sequencing of the same litter and soil samples used to characterize fungal communities^10^, bacterial community diversity metrics (richness, evenness) were measured to compare treatment-level differences in community composition at different taxonomic levels. Principal Coordinates Analysis (PCoA) was used to examine plot-level differences in bacterial families between treatments. Finally, influential bacterial families were identified that differed in relative abundance across factors in ways that drove broader community responses. These results provide a new microbial test for pyrodiversity and identify key bacterial taxa adapted to frequent, low-intensity fires to complement the growing research focused on post-fire adapted bacteria potentially important for ecosystem recovery and management^30,31^. The bacterial community shifts are divorced from variation in plants and edaphic properties, but may alter underlying ecosystem processes, including fuels, that feedback to future fires. This study also identified sensitive taxa that may be effective biological indicators of frequent fire regimes. With improved and cheaper techniques, these taxa may represent a useful management tool that provide a more foundational and comprehensive metric for system health. Finally, these results emphasize that those management regimes utilizing frequent prescribed fires also help conserve microbial diversity in fire-dependent ecosystems now fragmented from larger expanses once maintained by lightning-ignited fires^32^, especially highly biodiverse ecosystems like pine savannas^33^.

## Methods

### Field site and sampling of substrates

This study was conducted on the Wade Tract Preserve (30°45′ N; 84°00′ W). This preserve contains 85 ha of pine savanna/woodland located on moderately dissected terrain 25–50 m above sea level ~ 80 km north of the Gulf of Mexico in Thomas County, Georgia, USA (Supplementary Fig. S1, Supplement 1). Over the past couple of centuries, frequent fires^32^, coupled with no logging at this site, have maintained an open physiognomy with patches of old-growth overstory pines and diverse herbaceous-dominated ground layer vegetation (see photographs^23,34,35^). Fire return intervals of 1–2 years are possible because substantial rainfall (averaging ~ 1350 mm), and a 10–11 month growing season results in rapid post-fire regrowth of herbaceous ground layer plants that, coupled with abundant pine needles, generate sufficient fuel for burning of ground layer vegetation within a year^28,36^. These fires are of low intensity and do not burn the whole landscape, but leave many areas unburnt, thus patches may burn that were not burnt in previous fires, while burnt patches may be left unburnt. This generates the patchy nature of these frequent, low intensity fires. Further details of the Wade Tract Preserve, larger study site, and fire regimes are presented in Supplement 1 and in Semenova-Nelsen et al.^10^.

This study was established following two prescribed fires in the spring of 2014. Shortly after the fires, patches of the landscape that burnt and did not burn could be readily identified in the field by distinguishing ground layer vegetation and litter that had been burnt (i.e., charred), from vegetation and litter that remained intact and unaltered. For long-term study, unburnt patches of ground layer vegetation > 5m^2^ in size in a 50 ha upland pine savanna plot were established in 1978^27,28^. In 2014, these unburnt patches comprised 5–10% of the total 50 ha mapped plot. 30 naturally unburnt patches were then randomly selected from those mapped, 15 near and 15 away from pines, comprising pine proximity near and away, respectively. These unburnt patches were left naturally unburnt by the prescribed fire, without human intervention, and were selected subject to the constraint that they had burnt naturally at least once within the two prior years. Next, 30 burnt patches were randomly selected so as to pair (location within 5-10 m) each burnt patch with a nearby unburnt patch that had similar overstory pine proximity conditions. These burnt patches had naturally burnt in each of the past three years. Subsequently, one 1 × 1 m centrally located plot was installed inside each patch so that no plot edge was a border of the patch. A total of 60 total plots were used in this study (Supplementary Fig. S1, Supplement 1). More complete descriptions of plot selection are provided in Supplement 1.

Plots were sampled in mid-July following fires. Each vascular plant species present was recorded in each plot. Plant nomenclature followed Weakley^37^. Voucher specimens of each plant species included in the study were collected outside plots and deposited in the herbarium at Tall Timbers Research Station. Collection of plant material complied with institutional, national, and international guidelines and legislation. Specimens are in the Florida State University herbarium (https://herbarium.bio.fsu.edu/) under barcodes TTRS_000010437-TTRS_000010579. PC ORD 6^38^ was used to test plant community composition patterns related to burn and pine proximity treatments. Concurrently, three 9 × 9 cm areas were randomly sampled in each of the 60 plots, located so as not to require destruction of above-ground vegetation. In each area, two samples were collected, one of surface litter and one of surface soils directly below the litter to a depth of 1.5 cm. This is the depth to which temperature increases during fires are greatest^5,8^. All litter collection avoided recently fallen, post-fire material. The three samples for a given sample type (litter and soil) were pooled in separate sterile plastic bags. All sampling equipment was sterilized with 10% bleach and 90% isopropyl alcohol between plots to avoid cross-contamination. Samples were kept in a cooler with freezer packs, frozen at -20 °C within 4 h, and shipped overnight to the University of Kansas, where they were stored at -80 °C until laboratory analysis.

### Laboratory procedures

Each sample was thawed and thoroughly homogenized within the sealed collection bag. Following homogenization, approximately a 100 g subsample was used for chemical analyses, and a 2 g subsample was taken for soil or litter molecular analysis. Soil physical and chemical properties were assayed for each plot. The methods of analysis of soil samples and results of these analyses are presented in Supplement 2.

DNA was extracted and then amplified. 0.25 g of DNA was extracted from each 2 g subsample. MoBio PowerSoil Kits (MoBio, Carlsbad, USA) were used, and extracted DNA was quantified using Qubit 2.0 (LifeTechnologies, Carlsbad, USA). The V4 hyper-variable region of 16S rDNA was amplified from 5 ng of template DNA using the standard Earth Microbiome Primers 515F and 806R^39^ and Q5 polymerase (New England Biosystems, Ipswich, USA). PCR protocol consisted of an initial denaturing at 98 °C for 30 s; then 25 cycles of denaturing at 98 °C for 10 s, annealing at 55 °C for 30 s, and extension at 72 °C for 30 s; followed by a final extension at 72 °C for 5 min. PCR products were cleaned using Agencourt AMPure XP magnetic beads (Beckman Coulter, Indianapolis, USA). A second PCR was then used to ligate unique Nexterra indices (Illumina, San Diego, USA) to each sample. Cycling conditions for the indexing PCR were similar to the first PCR, except for only running for 8 cycles, and then purified as before. Individual libraries were pooled at equimolar concentrations into a single, 4 mM library; concentration and amplicon size was verified using a TapeStation 2200 (Agilent, Santa Clara, USA). Amplicon sequences were then generated using a 301 bp, paired-end run on an Illumina Mi-seq (Illumina, San Diego, USA) at the Kansas State Integrated Genomics Center. Sequencing data included a negative water-only library control (from 1st PCR step above) and 120 samples, generated from soil and litter in the 60 plots. Paired reads were concatenated, and Nexterra indices were used as barcodes for demultiplexing the sample reads from the 120 sequenced samples. One sample failed and was excluded from this study, to make a total of 119 samples plus one negative control.

Raw barcode reads were demultiplexed and assigned to exact sequence variants (ESVs). Reads were compared to the 119 possible barcode combinations and accounted for base pair read error to increase demultiplexing accuracy and identification rate. A maximum likelihood approach was used that assigned a test statistic from the geometric means of matched and unmatched barcode reads, weighted by Phred scores Z_Renaud_^40^. Z_Renaud_ accounts for greater number of N reads than Qiime and down-weights matches with high error, therefore recovering more samples than other approaches. To determine a Z_Renaud_ threshold for removal, 500 clearly denoted samples were identified that were missing 2–3 base pairs and were of similar barcodes with very high error probabilities. For this dataset, Z_Renaud_ > 6 maintained strong selectivity; so samples with a score of Z_Renaud_ ≤ 6 were removed. DADA2 was used for processing demultiplexed sequences following the big data script for paired end reads^41^. A truncation score of 2, maximum allowed errors of 3 and 5, and truncation lengths of 200 and 151 nucleotides for forward and reverse reads, respectively, were chosen.

ESVs were processed to further remove erroneous sequences. Since PCR amplification can result in erroneous reads in the order of 1:10^3^, rare ESVs were removed by using a less conservative threshold of 1:10^5^ for removal than often applied (1:10^4^)^42^ because type-I errors at the ESV level were not relevant to this study, and because erroneous reads could have been present in more common ESVs as well as more rare ESVs. Loss of ecological signal was minimized by avoiding arbitrary removal of correct reads. This threshold required an ESV to have a minimum total read count of ~ 70 over all samples to be part of the final dataset.

ESVs were then assigned taxonomy based on two known databases to maximize identification breadth and increase certainty. Identifications were compared using SILVA v132^43^ and RDP v11.5^44^ databases, which map well together^45^. These databases use the same species identification, but they use different systematics for higher taxa (family to class); SILVA was used for higher-order classification due to its greater size. Taxonomies were assigned hierarchically from kingdom to genus via a bootstrapping method that compared ESVs to database sequences where each lower level had increased chance of greater assignment error. All levels were output regardless of database match error and identified genera were compared. If genera agreed between both databases, that assignment and all higher-order SILVA taxonomies were used. Where genera did not agree, SILVA’s 80% threshold was used for identification to assign the lowest level included in analyses. This allowed substantially more classifications than SILVA alone.

Identified taxonomic groups were finally standardized into final datasets. ESVs were aggregated and standardized by taxonomic level (genus and higher) to examine relationships among identified taxonomic groups rather than ESVs directly. DeSeq2 was used to standardize consistent mean–variance relationships for each taxon among samples with assumed negative binomial distributions^46^. For overdispersed taxa, total reads were adjusted towards the mean to fit the overall mean relationship. Variation was removed in the substrate-fire treatment and then normalized within substrate-fire interaction to account for systematic read error between samples. To normalize reads to the mean, all taxa within each sample was divided by the geometric mean of positive counts. A value of 1 for a taxon meant that, in that sample, that taxon’s reads were exactly the mean; values X higher or lower than 1 denote that reads for those taxa are X times the mean of that sample. Thus, within a sample, these standardized data were approximately a 1:1 transformation of abundances, and presented relative abundances as multiplicative factors of taxon reads. Across samples, however, relative abundances of taxa were not 1:1 and could result in different results and model behavior. Total reads were estimates of genus-level total ESVs abundance per sample. Analysis used R package DeSeq2 v1.24^46^.

### Data metrics and analyses

Community-level analyses were conducted to relate data on bacterial taxa to experimental field treatments. Partial constrained principal coordinate analyses, hereafter PCoA^47^, was used. Analysis was conditioned on spatial variation effects, by using pairing of burnt/unburnt plots and certain soil properties, especially soil type as described in Supplement 2. Analysis was constrained on treatment effects of fire status (burnt/unburnt), substrate (litter/soil), pine proximity (near/away from overstory pines), and the interactive effects of these three experimental conditions. The dataset was first transformed via Hellinger transformation. Multiple PCoAs were then generated and compared on each taxonomic level to identify the taxonomic level that provided the greatest resolution of treatment effects. All PCoAs used Euclidian distances of the bacterial ESV response matrix. The family-level PCoA had the most proportion of variance explained by the combined linear combination and conditional effects, so family was selected as the taxonomic unit to measure relationships with experimental treatments. This family-level PCoA performed dimension reduction and presented two dimensions that represented the larger multidimensional site-taxa and explanatory variable relationships. Scores on the PCoA were then used to identify influential families, based on having absolute values of scores greater than the mean of the distance from the origin to interaction centroids on the PCoA. Influential families were families that strongly associated with the categorical treatment effects, and showed large differences in relative abundances across treatments, which resulted in larger scores on the PCoA, and thus identification as an influential family. Differences of influential family relative abundances, within each family, among treatments, were determined with the first generalized linear mixed model (GLMM). This first GLMM’s response variable was influential family relative abundances, and explanatory variables were the triple interaction of fire*substrate*taxa. This GLMM fit the triple interaction to a Tweedie distribution, a mixture of Poisson and Gamma distributions that accounts for large numbers of zeroes in count or continuous data and that can model data that have many zeros, but are otherwise Poisson distributed^48^. This first GLMM analyzed families simultaneously with a within-taxon standardization to the mean relative abundance of that family across treatments. This allowed inferences of relative abundance patterns for treatment versus treatment for any individual family, but did not allow comparisons of family versus family relative abundance patterns for any treatment. Due to this standardization, the families considered as part of this analysis occurred in all treatments at least once, but did not necessarily occur in all plots. A heatmap was then generated to visualize the influential family relative abundances tested in this first GLMM. Then, overall abundance and frequency of occurrence between influential and non-influential families were plotted to understand the relative impacts of influential families in the larger ecosystem. Further details of procedures used for PCoA analyses and the first GLMM for influential families are presented in Supplement 3. Further details of analysis of taxonomic levels and determination of family level use are provided in Supplement 4.

Beta diversity metrics were assigned on the family level over all plots in each treatment. Diversity metrics were assigned independently for each litter and soil sample in each plot. For each sample, differences in taxon richness and evenness were calculated among fire and substrate treatments using a set of additional GLMMs, one for each family-level diversity metric and treatment. Response variables were the beta diversity metrics, and explanatory variables were the main effects and interactions among fire treatments, substrate, and pine proximity, with all models including paired plots as random effects to match the experimental design randomly pairing burnt and unburnt patches. The GLMMs for richness were modeled as Poisson distributed with log link function. The GLMMs for evenness were modeled with a beta distribution and logit link because values of evenness were on the interval (0,1), but not binomial. Models were fit with mgcv 1.8 in R 3.5^49,50^.

PCoA results were also used in tests of beta diversity among and within treatment groups. Multivariate homogeneity of dispersion was tested via PERMANOVA to identify similarities among the groups by main effects and interaction of substrate*fire. Pine proximity was excluded as a variable due to its lack of effect in the set of GLMMs for family-level diversity metrics. As in the PCoA, the family matrix was first Hellinger transformed, then analyses used Euclidean distances.

## Results

### Bioinformatics

Sequencing produced 21,384,517 total reads, with a median read length of 309 nucleotides, and an average quality score of 33 (Phred-score). Following DADA2 and chimera removal, there were 7,084,243 total reads over 61,733 unique bacterial ESVs across 22 phyla. Following removal of rare ESVs and taxonomic assignment to produce the final dataset, there were 3,316,309 total reads over 7,675 ESVs, which comprised 178 genera, 83 unique families, 59 orders, 26 classes, and 13 phyla.

### Bacterial communities [partial constrained principal coordinates analysis (PCoA)]

**Figure 1:**
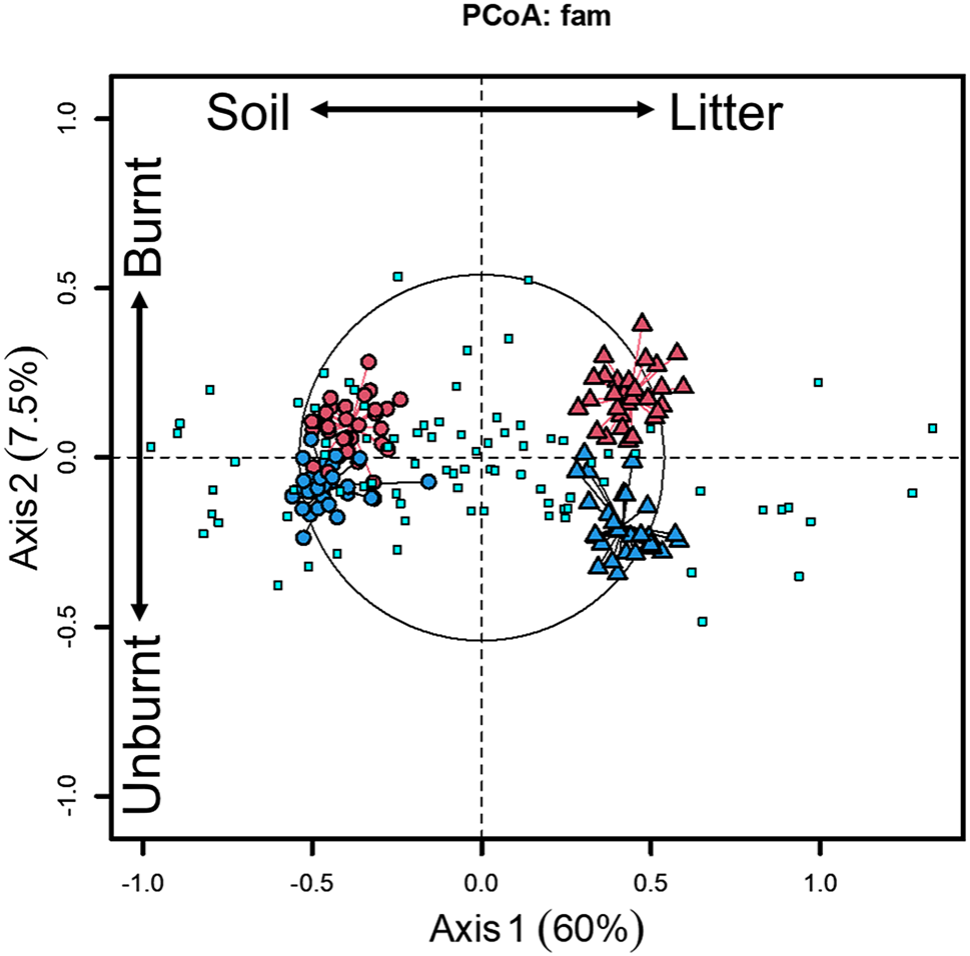
Principle Coordinate Analysis of plots driven by taxonomic family. First two axes of PCoA differentiated plots by community relative abundances, where increasing distances among points indicate greater differences between plots. Percent of total variance explained by each axis is given in parentheses. Labeled arrows parallel to axes indicate the associated treatment with the respective axis. Red points denote burnt plots and blue unburnt; triangles denote litter and circles soil. Cyan squares are each a family; large distances from the origin indicates influence on nearby treatment differences.

Experimental fires produced distinct bacterial communities, but this was not the sole, or even most important driver of bacterial community composition. Based on variation in relative abundances of families of bacteria, substrate, fire, and their interaction explained 67.5% of the total variation among samples (Fig. 1; marginal tests F_1,85_ = 126.0, 19.0, and 5.0, respectively; *p* < 0.001 for each). Substrate (axis 1) explained most of this variation (60% of the total, 89% of the constrained variation). Samples from litter and soil substrates were completely and widely separated along PCoA axis 1, with distances parallel to the axis. Fire, both as a main effect and through its interaction with substrate (axis 2), explained much of the rest of this variation (7.5% of the total; 11% of the constrained). Samples from burnt and unburnt litter and soil both were differentiated along PCoA axis 2, with distances parallel to the axis. Litter samples separated more completely than soil samples (Fig. 1), indicating that fire effects were greater in combusted litter than in the adjacent soil. The two conditioning effects of spatial variation evaluated through paired plots and soil type explained 20% of the total variation among samples beyond the constraining effects of treatment. The remaining 12.5% of total variation was unexplained by constraining or conditioning effects.

Several components of the field study (i.e., plant community composition, proximity to pines, soil properties) were not included in final family-based PCoA models. Pine proximity and plant community composition each had no significant effect (*p* > 0.05 for all comparisons) on bacterial community composition, and therefore they were not considered further in those analyses. Analysis of variance of numbers of vascular plant species in plots indicated no significant difference in plant species richness of burnt/unburnt plots, or near/away from pines (*p* > 0.05 for all comparisons). MDS ordination using PC ORD 6^38^ of plant species in plots did not indicate community patterns related to plots being burnt or not burnt. Some aspects of soils in burnt and unburnt plots differed (Supplement 2), but at least one burnt plot and one unburnt plot were each within the group of plots associated with the other fire treatment (Fig. 1). Soil properties often varied with one another (i.e., collinear) based on experimental fires, so they were examined only as main effects. Significant differences in % organic carbon, total nitrogen, and gravimetric water content (Supplement 2) all had weak effects on bacterial composition and reduced AIC values of bacterial PCoA models, so were not included in the final model.

### Influential families

Of the 83 bacterial families, 27 had significant (*p* < 0.001) effects in the first GLMM for influential families and were included in the final model. By contrast, the remaining 56 families did not strongly respond to treatment effects. Influential families were families that showed large differences in relative abundances across treatments and strongly associated with the categorical treatment effects, and thereby drove differences in the overall bacterial community composition visualized in the PCoA. These families thus influenced ordination on the PCoA. These families were distributed among different treatment combinations, as indicated by the spread of families in Fig. 1. The GLMM (Table 1) of influential families indicated significant main effects (family, fire, substrate, *p* < 0.001) and two-way interactions (family*fire, family*substrate, *p* < 0.001). Simultaneous 95% confidence intervals for each family indicated that significant differences resulted when one treatment mean was at least 1.25 times greater than another.

**Table 1:** Type-III tests of fixed effects from GLMM estimating relative means by treatment among influential families in PCoA community differences.

Effect	df	F	*p* value
Family	26	36.09	< 0.001
Fire	1	6.12	0.013
Substrate	1	63.26	< 0.001
Family*fire	26	9.8	< 0.001
Family*substrate	26	55.73	< 0.001
Fire*substrate	1	1.3	0.255
Family*fire*substrate	26	2.33	< 0.001

**Figure 2:**
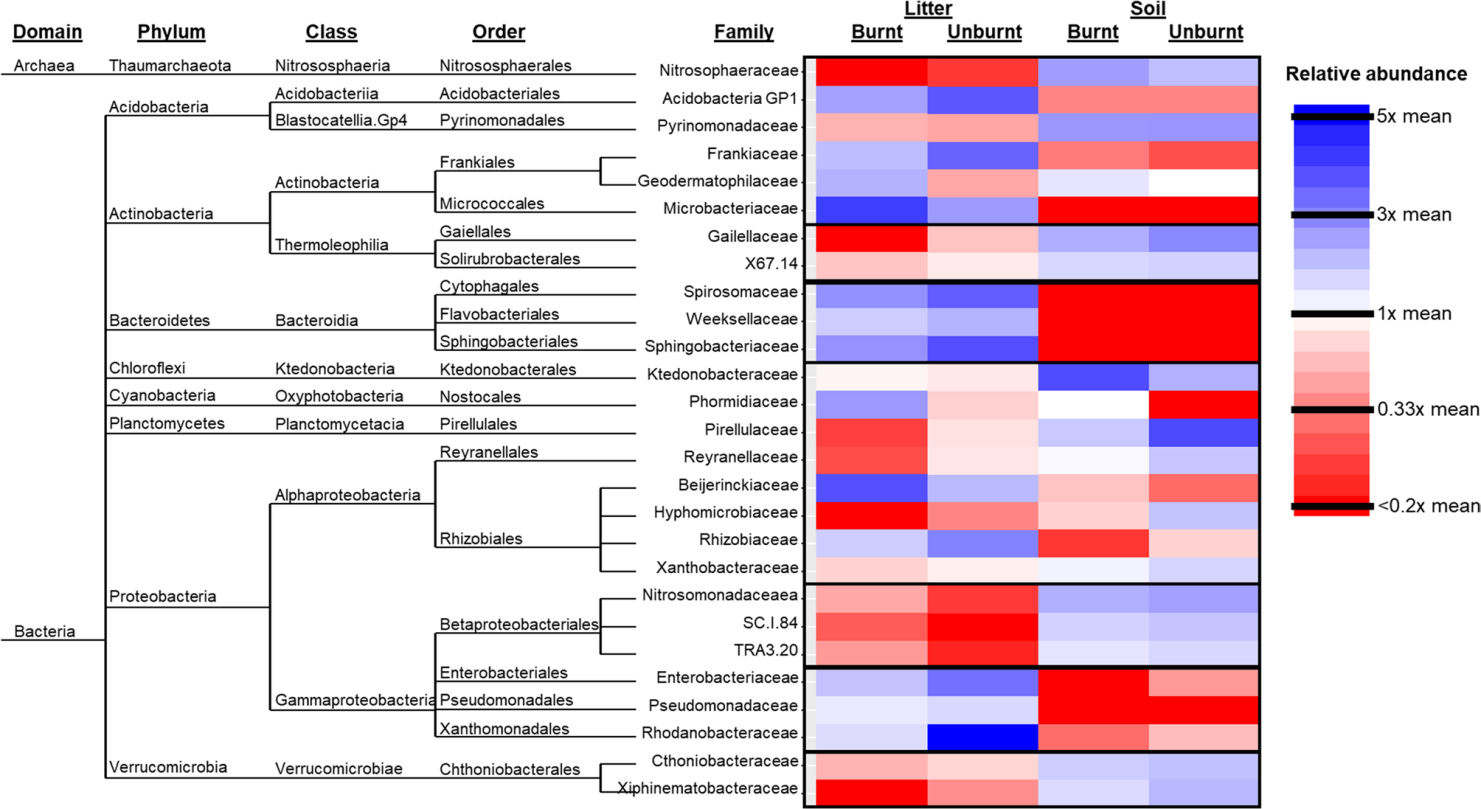
Phylogenetic tree and heatmap of influential families. The phylogenetic tree shows taxonomic relationships and higher taxonomic levels of the 27 influential families identified through higher PCoA scores. The heatmap depicts relative abundances, calculated as geometric means for each row and thus family, averaged over all plots in the treatment for that single family. Blue, red, and white shading indicates that family’s average relative abundance within a specific treatment being above, lower, or similar to, that family’s average relative abundance over all treatments, respectively. Black boxes highlight potential phylogenetic correlations of paraphyletic families showing similar patterns of relative abundances and treatment associations.

These 27 responsive influential families showed strong differences in mean-normalized relative abundances associated with treatments. Families overwhelmingly separated firstly by substrate and then secondly by fire (Fig. 1). Within substrate and often further between burnt and unburnt substrate, these families showed different relative abundances (Fig. 2) that supported results of the GLMM (Table 1). Regardless of fire treatment, 12/27 (12/27 = 44%) were litter specialists, which included Gammaproteobacterial families in the orders of Enterobacteriales, Pseudomonadales, and Xanthomonadales as well as all families in the class Bacteroidia. Also 15/27 (55%) were soil specialists (unrelated to fire). These included all three families of the order Betaproteobacteriales (Gammaproteobacteria), both families in class Thermoleophilia, and two families in the phylum Verrucomicrobia. The majority of families showed a general substrate*fire association. Nonetheless, many of these substrate*fire associations were relatively weak, as many families associated primarily to substrate and appeared to be much less affected by fire treatment. Only Geodermatophilaceae and Phormidiaceae showed primary association to fire (unrelated to substrate) with higher abundance in burnt compared to unburnt substrate. Members of the family Beijerinckiaceae specialized on burnt litter, while the families Rhizobiaceae, Enterobacteriaceae, and Rhodanobacteraceae were associated with unburnt litter. Only Ktedonobacteraceae was somewhat associated with burnt soil, while the members of the families Pirellulaceae, Reyranellaceae, Hyphomicrobiaceae, and Xiphinematobacteraceae, comprising 4/27 (15%) of families, were all associated with unburnt soil.

Some phylogenetic correlation was present relative to substrate. Influential families were occasionally part of paraphyletic clades showing similar associations (Fig. 2). Within the class Gammaproteobacteria, for example, the order Betaproteobacteriales (all three families) was strongly associated with soil; the other three orders Enterobacteriales, Pseudomonadales, and Xanthomonadales (each with a single family) had strong associations with litter. All orders in the class Bacteroidia were associated with litter, while class Thermoleophilia was soil-associated. The two families in phylum Verrucomicrobia were both soil-associated.

**Figure 3:**
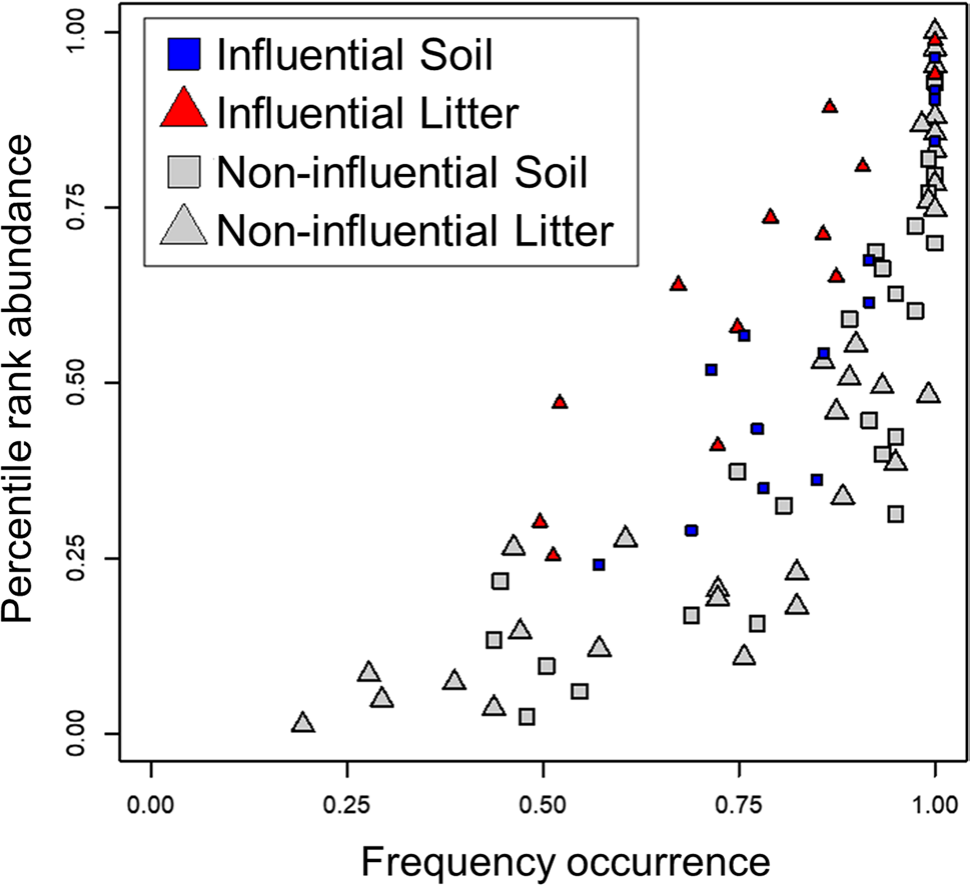
Family abundance and occurrence. Frequency occurrence refers to the percentage of how many plots that family was found in. Percentile rank abundance refers to the rank abundance of that family over all plots. Blue squares denote influential families generally associated with soil, red triangles with litter. Grey squares and triangles denote non-influential families associated with soil and litter, respectively.

Influential families exhibited unexpected patterns of abundance and occurrence that emphasized the impacts of more rare families. The 27 families tended to show moderate abundance, but reduced occurrence in plots compared to non-influential families (Fig. 3). There were no differences in mean abundances or occurrences between groups (t-tests; *p* = 0.246 and 0.809, respectively). Influential families were widely variable in their abundance, as the least abundant family was at the 0.25 percentile abundance, greater than 19 other families, but otherwise influential families were among the top 3 quartiles of abundance. Influential families were by far not the most frequently occurring families either, as only 6/32 (19%) families that occurred in > 90% of plots were influential. Most were not found in all plots, as 21/27 (78%) influential families had proportional occurrence 0.49–0.91 while 6/27 (22%) were found in all plots.

### Beta diversity

**Figure 4:**
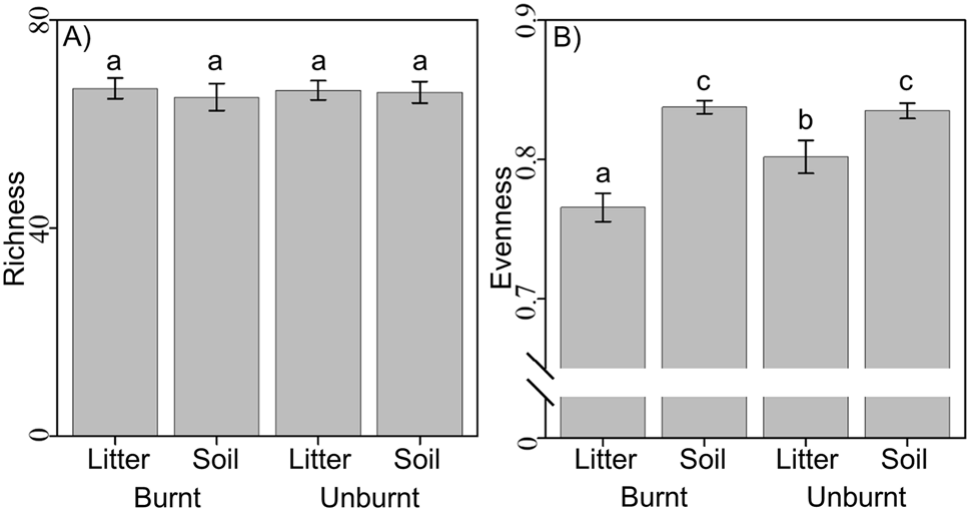
Comparison of family-level diversity metrics. Metrics are presented among fire and substrate treatments for: (**A**) richness, and (**B**) evenness. Same letters denote group memberships that are significantly different than other letters. Shown are empirical geometric means with 95% confidence intervals.

On the family level, treatments showed broad patterns in richness and evenness that were congruent with PCoA results. Neither plant community composition nor pine proximity strongly affected richness or evenness of bacterial communities. Bacterial family richness was almost identical among treatments (Fig. 4A), with combined geometric mean 66 $\frac{*}{/}$ 1.02 (66 multiplied and divided by 1.02). For evenness, main effects of fire and substrate, and their interaction were all highly significant (*p* < 0.0001, Table 2). Family relative abundance was more even in soil than in litter, regardless of fire treatment, and was less even after fire in litter samples (Fig. 4B). Particularly, soil evenness varied less and did not differ between fire treatments (geometric mean 0.84 $\frac{*}{/}$ 1.003), while bacterial relative abundance was more even among unburnt than burnt litter samples (0.80 $\frac{*}{/}$ 1.008 and 0.77 $\frac{*}{/}$ 1.007, respectively). Soils have higher Shannon diversity than litter. Among and within treatment groups, dispersion was homogeneous over groups, indicating no significant differences within groups (F_3,115_ = 0.8; *p* = 0.514). There were significant beta diversity differences among groups (F_3,115_ = 64.5; *p* = 0.002), for both main effects (substrate and fire, each P_1,115_ = 171.4, *p* = 0.002), and substrate*fire interaction effects (F_1,115_ = 4.7, *p* = 0.015).

**Table 2:** Type-III test of fixed effects for richness and evenness GLMMs.

	Richness		Evenness	
Effect	${\varvec{\chi}}_{1}^{2}$	*p* value	${\varvec{\chi}}_{1}^{2}$	*p* value
Fire	0.037	0.847	70.78	< 0.001
Substrate	0.532	0.466	224.24	< 0.001
Fire*substrate	0.165	0.684	41.81	< 0.001

## Discussion

This study revealed resilient, taxon-rich, biodiverse bacterial communities inhabiting ground level litter and soil substrates, which complement the high plant diversity that is characteristic of the old-growth pine savanna ecosystem on the Wade Tract^23^. The effect of low intensity fire reorganized the bacterial communities in both litter and soil, and shifted relative abundances but did not affect diversity. Study of the fungal communities in this same ecosystem revealed a similar reorganization^10^. Frequent, low intensity fires appear to maintain biodiversity not only of plants, but also both bacterial and fungal communities in this fire-adapted pine savanna ecosystem^51^.

### Localized within site differences, bacterial communities were driven primarily by substrate heterogeneity and low intensity fire effects, not specific effects of plant or soil variables

The Wade Tract’s history of frequent, patchy, low intensity fires generates and maintains substrate heterogeneity that drives bacterial communities. Firstly, in the absence of fire, substantial differences between substrate types generated complex heterogeneity associated with distinct litter and soil bacterial communities. These differences are tied to chemical and physical substrate differences, including available compounds^52^, decomposition stage^53,54^, C:N ratios^55^, and substrate quality^56^. Secondly, fire also altered the substrate heterogeneity, providing both direct and indirect effects that further differentiate litter and soil communities. The greater distinction between burnt and unburnt litter bacterial communities compared to soil is likely attributed to direct exposure of litter and associated bacterial communities to fire effects^57^. Indirect effects related to post-fire litter chemistry, partial combustion (charring) of fuels^58^, the various decomposition stages of post-fire standing litter^58,59^, and the abiotic changes in litter properties over time since fire^60^ may also play a secondary role^8^. In contrast, soil insulating properties may also reduce fire effects on soil communities through direct^61^, or indirect effects^15^. Compared to direct effects, indirect fire effects may be especially impactful on soil and litter bacterial communities in ecosystems affected by frequent, low intensity fires, and may be more pronounced for soil bacterial communities than those in the litter^60^. Substrate had a larger effect than fire, but results support two hypotheses: bacterial communities should differ between fire treatments, and the differences should be greater for litter than soil.

The fire-induced differences in bacterial communities were not explained by differences in plants or soil variables. Plants directly affect bacterial communities via litter decomposition and belowground rhizosphere deposition^62^; and indirectly by plant effects on soil properties^63^. Changes in plant communities and soil variables have been linked elsewhere to bacterial community changes^64^. Fire frequency and time since fire may be important mediators of plant and soil effects on bacterial communities in ecosystems affected by frequent, low intensity fire^60^. More intense fires more strongly disturb plants^61^ and soil variables^65^ to produce indirect, severity-linked effects on bacterial communities^66^. In this system however, lower intensity fires did not strongly affect plant communities and thus may have negated clear, measurable fire-associated plant effects on bacterial community composition or relative abundances. Localized pine proximity effects on bacterial communities were not apparent, despite increases in fuels and fire intensity near overstory pines^29^. A combination of frequent fires, high plant litter diversity^67^ throughout the site, and fresh pine needle inputs combined with functional overlap across pine decomposition stages^68^ may all have played a role muting the localized differences predicted. Although these fires did alter key soil properties, these edaphic shifts were not linked to bacterial community differences. This lack of effect may be a result of low fire intensity and frequent fire regimes^69^, rapid post-fire recovery of altered soil variables^70^, or the initial soil or litter effects diminished with time since fire^71^. A reduced importance for soil properties compared to site-level characteristics and soil layers has also been shown in Wisconsin pine barrens^72^ and so may be a common feature of these systems. Ecosystems with more severe and/or less frequent fires are likely to still have bacterial community differences among substrate types, but will also have greater fire-induced changes to plants and soil variables than seen here, and these shifts may be more readily tied to responses of bacterial communities to fire^73^. Bacterial shifts, however, do not appear to depend on fire-induced plant or soil changes alone, and neither on localized fire effects associated with pine proximity. These findings did not support the hypothesis of differences in bacterial communities due to localized fire effects related to pine proximity.

Frequent, low intensity fires are important in maintaining bacterial community diversity and variability in this savanna ecosystem. Despite having distinct communities, richness was nearly equal across substrate and fire factors; thus these frequent, low intensity fires help maintain distinct community components on the landscape by reorganizing bacterial taxa without causing large changes in composition and relative abundances of bacterial taxa in these communities. In agreement with pyrodiversity literature^51^, these results suggest that low intensity fires at the Wade Tract maintain bacterial biodiversity, including, fire-agnostic, fire-sensitive, and fire-dependent taxa^51,74^. This work supports others who found that frequent, low intensity fire is important for maintaining bacterial community diversity in African woodland^75^, Australian wet sclerophyll^76^, Chinese pine^57^, and Oklahoman oak forests^77^. Fire severity, however, is critical to generalize these results to other ecosystems, as different fire types and intensities alter soil variables differently than litter variables, and result in post-fire soil environments with varying magnitudes of change^78^. Lower intensity fires in grasslands and savannas often leave a heterogenous litter environment of unburnt, burnt, partially combusted, and fresh, new litter^5,79^. Higher intensity fires result in deeper heat penetration^80^ that result in stronger sterilization effects on bacterial communities^81^. By contrast, lower intensity fires produce heating effects that do not penetrate as deeply or strongly, and thus do not result in homogenous post-fire soil substrate environments^82^. Maintaining substrate heterogeneity via frequent, low intensity fire is likely important to maintain larger microbial biodiversity on long-term scales, which affect microbial ecosystem functions such as decomposition, nutrient cycling^83^, and plant interactions^13^ and plant recovery^84^ that are important to consider for adaptive fire management^60,85^. This is especially important in consideration of changes in fire regimes related to human and climate effects^24,25^. The biodiversity of bacterial communities should be a characteristic of old-growth fire-frequented ecosystems^23^ that complements the large plant diversity characteristic of this and other pine savanna biodiversity hotspots^33^. Frequent, low intensity fires appear to simultaneously maintain all ground layer components of these firey ecosystems, including the substrate heterogeneity and biodiversity in the plant and microbial communities.

### Bacterial communities differed in the relative abundances of particular, responsive, influential families associated with treatments

27 distinct families were identified whose abundances were clearly linked to differences in substrate, fire, and their interactions. These differences are likely driven by niche differences among bacteria^71^, and their filtering by the heterogeneity that substrate and fire generate^86^. Interestingly, all the influential families occurred in all treatments, and richness was not different even when abundance differences indicated preferences^87^. Bacterial dispersal may be high, particularly if many are spread through updrafts and smoke^88^. Even in “unpreferred habitats,” bacteria may still be represented by background dispersal, resting stages, or even post-fire relic DNA that is indistinguishable from viable microbes with the tools used here^89,90^. Given the ample rainfall, poor nutrient soils, and repeated fires, however, relic DNA was not expected to bias the evaluation of substrate and fire effects overall^90^. Bacteria that were much more abundant in specific substrates, or substrate*fire combinations likely reflect substrate preferences including those found only in stages of litter decay^68^ or soils^91^. The taxonomic and phylogenetic signal for these associations, particularly between substrates, supports the role for environmental filtering of functionally similar groups^92^. For example, Beijerinckiaceae were strongly associated with burnt litter. Other work have shown them enriched by partially combusted organic matter^93^ and nutrient pulses^94^, and responsible for C1 (1-carbon) metabolism^95^ including carbon monoxide. Similar identification of potentially fire-adapted microbes across ecosystems may provide bioindicators of post-fire response. For example, consistent with others’ findings of the enrichment of Blastococcus in burnt soils^73^, its family Geodermatophilaceae was found here to be associated and enriched in both burnt litter and soil substrate. The preferences by specific groups may be a consequence of fire-induced substrate heterogeneity, and further catalyze specific changes in ecosystem processes given their functional roles in nutrient cycling and decomposition^83^. Future work will investigate potential functional changes in the bacterial community correlated with differences in influential family relative abundances, and how changes in bacterial community function may affect ecosystem management.

Differences in overall microbial decomposition seen previously are likely a product of combined differences in bacterial and fungal communities. Using size-selective mesh bags (38 µm), Semenova-Nelsen, et al.^10^ found that new fuels in these burnt plots decomposed at a rate more than 30% slower than those in the unburnt plots. The bacterial community differences mirror those of fungal communities, and both groups could enter through these mesh openings. Bacteria that specialize on burnt litter (e.g., Beijerinckiaceae) may be much poorer decomposers than those families that dominated communities of unburnt litter (i.e., Rhizobiaceae, Enterobacteriaceae, and Rhodanobacteraceae), and their suppression with fire may help explain the slowed decomposition. Importantly, a majority of bacterial families did not respond to treatments, whereas a majority of fungal OTUs responded in some way^10^. This likely reflects that many dominant or frequently occurring bacterial species are either fire-resistant or fire-adapted^58^, or are more resistant and resilient than fungi^19,31^. Given differences in life history, faster bacterial growth rates after fire should let them rebound more quickly than fungi and so they may play a greater role in ecosystem processes just after fire. Nevertheless, the responses of these two foundational groups, bacteria and fungi, are likely to also affect the rates of fuel accumulation in ways that may help future fires spread on the landscape. Future studies that can identify fire-adapted taxa and the core microbiome of this ecosystem^96^ can expand knowledge about fire-adaptations, and aid prescribed fire management. Their postfire responses affect nutrient cycling, decomposition, and post-fire plant growth and recovery^13,84^, and can serve as effective bioindicators of larger ecosystem health, response, and recovery^30,31^.

## Conclusions

Frequent, low intensity fire generates substrate heterogeneity (i.e. pyrodiversity) that is critical for maintaining diversity of not only fire-adapted bacterial, but also fungal and plant communities in the larger fire-adapted savanna ecosystem. While many families appear fire-resistant, responsive families tend to be restricted to specific substrates (litter/soil) and sometimes only in unburnt or recently burnt litter. In contrast to more intense fires, these frequent, low intensity fires do not strongly affect either plant communities or soil variables, which here were not linked to observed shifts in bacterial communities. This independence means bacterial components are not simply products of soil and plant fire responses. Additionally, lower intensity fires bring nuanced effects on influential families that contrast with more extreme changes in bacterial communities observed in response to high intensity wildfires. The high plant and fungal biodiversity known in this system is also present in the bacteria, highlighting the importance of continuing ecologically meaningful frequent-fire management. Moreover, the bacteria that either resist or are even enhanced directly after fire likely contribute to ecosystem processes like decomposition, which can feedback to alter fuel loads and future fire regimes. Future studies should explore the functional roles of these fire-adapted communities, including whether a core microbiome exists and whether the postfire bacteria act to maintain unique successional trajectories (e.g., priority effects). These microbiomes potentially can serve as effective bioindicators of fire-dependent systems and may represent key taxa needed for restoring these endangered, and biodiverse ecosystems. Fire-adapted ecosystems such as grasslands and savannas hold global roles in biodiversity, ecosystem services, and functions, but are declining due to synergizing effects of human and climate change effects. Maintaining these important ecosystems through prescribed fire management relies on considering biodiversity of not just the plant, but also the microbial communities that affect resilience and recovery.

## Supplementary Information

Supplementary Information 1.

Supplementary Figure S1.

Supplementary Figure S2.

Supplementary Figure S3.

## Data Availability

Multiplexed .fastq sequencing data are available in the National Center for Biotechnology Information Sequence Read Archive, accession no. PRJNA858493. The entire bioinformatic pipeline is available on request. Voucher specimens of each plant species included in the study were deposited in the herbarium at Tall Timbers Research Station. These voucher specimens are also accessible in the Florida State University online herbarium (https://herbarium.bio.fsu.edu).
