# Supplementary material for: Substrate and low intensity fires influence bacterial communities in longleaf pine savanna
Source: Sci Rep. 2022 Dec 3;12:20904. doi: 10.1038/s41598-022-24896-x (PMC9719495; doi:10.1038/s41598-022-24896-x)
Supplement: Supplementary file 1 — Supplementary Information 1. [file 41598_2022_24896_MOESM1_ESM.docx]

**Supplement 1 – Field Site Description**

The Wade Tract contains exemplary old-growth pine savanna/woodland habitat. The species-rich vegetation appears typical for uplands within the southeastern coastal plain of eastern North America^[1]^. These habitats contain patches of overstory trees dominated by longleaf pine, *Pinus palustris*, with a diverse midstory of scattered hardwoods, especially species of *Quercus* and *Carya*^[2]^. The ground layer contains more than 500 species of vascular plants; these include warm season grasses (e.g., *Aristida, Sorghastrum, Schizachyrium, Andropogon*), as well as large numbers of forbs and shrubs, almost half of which are endemic to pine savannas of the North American Coastal Plain biodiversity hotspot^[3]^. Soils are Ultisols (Typic and Arenic Kandiudults) with sand or sandy loam A and E horizons and sandy clay loam Bt subhorizons derived from Pliocene sediments of the Miccosukee Formation^[4-6]^. The site is protected by a conservation easement held by Tall Timbers Land Conservancy.

The Wade Tract is managed using frequent ground layer fires. Annual-biennial fires are known to have occurred since the early 1800’s^[7]^. Each of two burn units comprising the preserve is imbedded in larger areas within which prescribed fires have been conducted since 1978, when the easement was instituted. Records indicate 60 prescribed fires during the 4 decades between 1980 and 2020, 90% of which occurred between mid-March and late June, with return intervals averaging 1.5 years. These frequent fires, coupled with no logging, have maintained an open physiognomy with patches of old-growth overstory pines^[6,8-10]^ and diverse herbaceous-dominated ground layer vegetation.

We established our study following two prescribed fires conducted in late March and early May 2014. Drip torches ignited head/flanking fires along each burn unit boundaries 7-14 days after rain in mid-late morning, with winds of 10-20 km/hr and relative humidities of 50-60%. Flame fronts in the ground layer vegetation were 1-2m in height, and fine fuel consumption varied from 60-90%. These fires burned patchily, such that ~10% of the Wade Tract was unburned in scattered patches ranging in size from a few square meters to about half a hectare^[6]^.

**Supplement 1 – References**

1 Peet, R. K., Platt, W. J. & Costanza, J. K. in *Ecology and Recovery of Eastern Old-Growth Forests* (eds Andrew M. Barton & William S. Keeton) 39-62 (Island Press/Center for Resource Economics, 2018).

2 Noel, J., Platt, W. J. & Moser, E. Structural Characteristics of Old‐ and Second‐Growth Stands of Longleaf Pine (Pinus palustris) in the Gulf Coastal Region of the U.S.A. *Conservation Biology* **12**, 533-548, doi:10.1111/j.1523-1739.1998.96124.x (1998).

3 Noss, R. F. *et al.* How global biodiversity hotspots may go unrecognized: lessons from the North American Coastal Plain. *Diversity and Distributions* **21**, 236-244, doi:<https://doi.org/10.1111/ddi.12278> (2015).

4 Lawton, D. E. M., W.E.; Moye, F.J.; Murray, J.B.; O’Connor, B.J.; Penley, H.M.; Sandrock, G.S.; & Friddell, M. S. H., J.H.; Huddlestun, P.F.; et al. (Georgia Department of Natural

Resources and Georgia Geological Survey, Atlanta, GA, USA, 1976).

5 Sanders, T. E. *Soil Survey of Leon County, Florida*. 151 (US Department of Agriculture, Soil Conservation Service and Forest Service, 1981).

6 Robertson, K. M., Platt, W. J. & Faires, C. E. Patchy Fires Promote Regeneration of Longleaf Pine (Pinus palustris Mill.) in Pine Savannas. *Forests* **10**, doi:ARTN 367

10.3390/f10050367 (2019).

7 Rother, M. T., Huffman, J. M., Guiterman, C. H., Robertson, K. M. & Jones, N. A history of recurrent, low-severity fire without fire exclusion in southeastern pine savannas, USA. *Forest Ecology and Management* **475**, 118406, doi:<https://doi.org/10.1016/j.foreco.2020.118406> (2020).

8 Platt, W. J., Evans, G. W. & Rathbun, S. L. The Population Dynamics of a Long-Lived Conifer (Pinus palustris). *The American Naturalist* **131**, 491-525 (1988).

9 Platt, W. J. & Rathbun, S. L.

10 Mugnani, M. P., Robertson, K. M., Miller, D. L. & Platt, W. J. Longleaf Pine Patch Dynamics Influence Ground-Layer Vegetation in Old-Growth Pine Savanna. *Forests* **10**, 389 (2019).

**Supplement 2 – Soil Properties Analyses and Results**

**Supplement 2 – Methods**

*Soil laboratory analyses.* For each plot, soil properties were assessed using physical and chemical analyses conducted at the University of Kansas and the Kansas State University Soil Testing Laboratory. Soil particle size was measured following USDA standard field procedures, derived from the Wentworth scale, using a modified hydrometer method ^[11]^. Soil pH was measured using 1:2 (v/v) soil: ddH_2_O solution using pH-meter (Mettler Instruments, Toledo, USA). Gravimetric soil moisture (%) was measured as weight loss after drying a 10g soil subsample at 60°C for 48 hours, then soil organic content (%) was measured by sample ignition at 550°C for 1 hour. Phosphorus content (ppm) was measured using the Mehlich-3 method on a Lachat Quickchem 8000 (Lachet Instruments, Loveland, USA). Nitrogen (%) was analyzed using a LECO TruSpec CN Carbon/Nitrogen combustion analyser (LECO Corporation, St. Joseph, USA).

*Soil property response variables.* For each soil property, we calculated two response values: 1) Z-transformed values for different soil properties across all plots, and 2) ratios of unburnt to burnt values for each of the paired plots. These variables were complementary: Z-transformed values indicated experiment-wide measurement differences among plots, and ratios indicated differences among paired plots. Explanatory variables for each model of soil property included effects of fire (burnt/unburnt), and proximity to pines (near and away).

Sample raw data indicated that soils tend to be spatially sorted across the Wade Tract. We ran a principal components analysis (PCA) on soil physical composition; the ratios of sand, silt and clay, which sum to one, were highly correlated. We used the first principal component (79% explained variance) as a new variable “soil type,” which was positively correlated to sand and negatively correlated to silt. Therefore, we included soil composition as a random effect in models to address spatial autocorrelation of residuals.

Both models included paired plots as a random effect to account for local covariance among properties. Because residual distributions for both response variables were highly right skewed, but otherwise Gaussian, we fit both response variables to t-distributions with identity link using generalized additive mixed models (GAMM) in package mgcv v1.8-28 in R v3.6.0 ^[12-14]^. Random effects applied parametric ridge regressions on interactions among random slopes and intercepts by taking advantage of the link between smooth functions and random effects ^[15]^. Residual diagnostics for both models showed normally distributed residuals with good quantile-quantile fits between predicted values and response.

*Values of soil properties.* We estimated experiment-wide mean fire and pine effects on each soil property by fitting a model to the Z-transformed standardized values for each soil property across all plots. We regressed Z-values on main effects plus 2-way interactions of soil properties with pine proximity and fire (*Property + Pine + Fire* + *Property*Pine + Property*Fire*). Interactions with pine and fire effects were not included as fixed because they produced models with inflated variances; random effects included pairs and the soil type principal component, both grouped by *Fire*Pine* interaction and fitting random intercepts and slopes, respectively. Phosphorus distributions were both highly skewed and variable, necessitating removal of high values (>60 ppm) before Z-transformations to maintain homogeneity of variance among effects.

We used *a priori* contrasts to test for differences in mean values for fire/no fire and pine proximities for each soil property. As there was no fixed effect interaction between treatment variables, we did not test for interactive effects of these treatments. We predicted that fire would, in all burnt plots, volatilize and thus reduce nitrogen and organic carbon; we also hypothesized that burnt plots would have soils exposed to the air and thus and gravimetric water content would be reduced. Thus, we used 1-tailed tests that soil property values would be on average greater in unburnt than burnt plots. We had no *a priori* predictions for fire effects on pH, phosphorus, and carbon:nitrogen ratios, or on any pine differences, so we used 2-tailed tests. While we reported and graphed back-transformed response means and 95% confidence intervals, all contrasts were on Z-transformed values. Tests were only conducted within each soil property. Tests were orthogonal among soil properties, but were not within each soil property; therefore, we applied Bonferroni adjustments to the two tests within each property only.

*Ratios of soil properties in unburnt/burnt plots.* We estimated plot-level fire and pine-proximity effects among soil properties by fitting a model to the standardized ratios of unburnt to burnt values between paired plots. Ratios greater than 1 denoted smaller values in burnt plots. We regressed the ratios on main effects and interactions between soil property and pine proximity (*Property + Pine + Property*Pine*). Ratios were nonlinear relative to unburnt values (response numerator); we therefore Z-transformed the unburnt values within each soil property across all pairs and included the Z-values as a control variable in the triple interaction *Z*Property*Pine*. Z-transforming the unburnt properties similarly scaled the values and decoupled implicit correlations to the response. Random effects included plot pairs, plus the interaction between the soil type principal component of both unburnt and burnt plots, both grouped by pine proximity and fitting random intercepts and slopes, respectively. Phosphorus distributions were both highly skewed and variable, necessitating removal of high values to maintain homogeneity of variance among effects. Therefore, before Z-transformations, we removed phosphorus values above 120 ppm in unburnt plots for modeling ratios.

We used *a priori* contrasts to test for fire and pine treatment effects for each soil property, separately. We predicted that fires would volatilize nitrogen and organic carbon, thereby reducing concentrations in burnt relative to unburnt paired plots; we expected burnt plots to lose soil surface water by evaporation, thereby reducing concentration relative to paired unburnt plots. Thus, we used 1-tailed tests that the ratios within each pine proximity treatment were >1. We had no *a priori* predictions for pH, phosphorus, and carbon:nitrogen ratios, so these were 2-tailed tests. We tested pine proximity effects by comparing ratios between near/away pine treatments; all tests were 2-tailed. For fire effects, we calculated each ratio at the pine-specific geometric mean to account for both skewed response distributions and any differences in unburnt values between proximities. For pine proximity effects, we calculated ratios at the overall mean between near/away from pines to test for differences independent of concentrations. Tests were conducted within each soil property only. Among properties, tests were orthogonal but were not within each soil property; therefore, we applied Bonferroni adjustments to the three tests within each soil property only.

**Supplement 2 – Results**

*Values and paired ratios of soil properties.* The soil property values model estimated overall means that measured landscape-level variation, and the unburnt/burnt ratios of each soil property in paired plots estimated pairwise plot-level variation. Estimates of values and ratios largely agreed with each other on specific effects of treatments. When compared to each other, the models suggested that some soil properties relate more to plot-level variation and some more to landscape-level variation. Estimates of values (Supplementary Tables S2-1, S2-2, S2-3; deviance explained 29.6%) suggested significant effects of prescribed fires (Supplementary Table S2-1; P = 0.0007) and proximity to pine effects (Supplementary Table S2-2; P = 0.0027) among soil properties. Due to lack of interaction between fire and pine treatments, effects of each treatment were constant across the other; we show the additive effects of overall mean fire and pine effects combination with confidence intervals for each property (Supplementary Table S2-3; Supplementary Fig. S2). The unburnt/burnt ratios (Supplementary Tables S2-4; deviance explained 40.1%) indicated significant plot-level differences in pairwise fire effects within and between proximity to pines among soil properties controlling for Z-transformed unburnt values (P < 0.0001).

Treatments affected some physical properties of soil on the Wade Tract. Soil acidity was constant across plots (Supplementary Fig. S2). Overall average pH values were both nonsignificant for prescribed fires and pine effects, as were pairwise proportional unburnt/burnt differences within and between plots differing in proximity to pines. Prescribed fires dried out plots, regardless of pine proximity (Supplementary Fig. S2). Overall, unburnt plots had 1.33 times the water content of burnt plots, with an average reduction of 5.87% saturation among plots; water content did not differ between pine treatments. Pairwise ratios indicated that unburnt plots were 1.17 times wetter than burnt plots, both near and away from pines. These ratios suggest that pairs vary similarly in wetness, indicating that pairwise soil moisture values vary more at the landscape-level than locally between paired plots.

Treatments also affected chemical properties of soil on the Wade Tract. Phosphorus, alone among soil properties, was highly variable across the landscape and spatially correlated among plots (Supplementary Fig. S2). Landscape-wide, fires did not affect phosphorus values; however, the pine proximity effect was pronounced, with average phosphorus levels 29.2 ppm away from pines and 17.3 ppm near pines, a proportionate difference of 1.68. At the plot-level, there was also no effect of prescribed fires away from pines; however, the unburnt/burnt ratio of phosphorus near pines was 0.77 times that away from pines. Given the highly significant pairwise increase in phosphorus in burnt plots near pines with a confidence interval that overlaps ratios away from pines, it is possible that that possible that the nonsignificant result away from pines is a type II error. Further study on phosphorus should control for spatial distribution.

Prescribed fires reduced nitrogen levels similarly for both pine treatments (Supplementary Fig. S2). Overall mean levels in unburnt plots were 1.25 times the levels in burnt plots, a decrease following prescribed fires of 0.15% to 0.12%. Pairwise ratio compared to no effect of 1 suggested similar fire effects locally and landscape-level. No differences were evident between near/away from pines in either levels or ratios.

Organic carbon levels were reduced by prescribed fires overall (Supplementary Fig. S2). Mean levels were 1.18 times higher in unburnt plots, decreasing from 10.0% to 8.5% where burnt. Levels did not differ between pine treatments. Accounting for local variation, pairwise ratios were not significant near pines, but were 1.27 times higher away from pines. The negative results near pines may be type-II error that reflects heterogeneously distributed litter within pairs at the plot level or also greater woody fuels near pines. Future examination should control for litter composition.

Overall carbon:nitrogen ratios were higher near pines than away from pines (Supplementary Fig. S2). The mean value of the C:N ratio away from pines was 0.89 times that near pines. In contrast, there were no pairwise effects on unburnt/burnt ratios. These results suggest that on average nitrogen might be reduced by prescribed fires relatively more than organic carbon. However, comparing among confidence intervals, the positive result between pines suggests a possible type I error consistent with similar proportionate reductions in both treatments.

We caution that the relatively low deviance explained by the values model reflects high spatial correlation among properties. The Z-transformed unburnt covariate removes apparent residual autocorrelation, but it does not explain spatial relatedness. Because spatial components do not change the overall fire and pine relationships, we used the simpler model.

**Supplement 2 – References**

1 Bouyoucos, G. J. Hydrometer Method Improved for Making Particle Size Analyses of Soils. *Agronomy Journal* **54**, 464-465, doi:<https://doi.org/10.2134/agronj1962.00021962005400050028x> (1962).

2 Wood, S. N. Fast stable restricted maximum likelihood and marginal likelihood estimation of semiparametric generalized linear models. *Journal of the Royal Statistical Society: Series B (Statistical Methodology)* **73**, 3-36, doi:<https://doi.org/10.1111/j.1467-9868.2010.00749.x> (2011).

3 Wood, S. N., Pya, N. & Säfken, B. Smoothing Parameter and Model Selection for General Smooth Models. *Journal of the American Statistical Association* **111**, 1548-1563, doi:10.1080/01621459.2016.1180986 (2016).

4 Team, R. C. R: A language and environment for statistical computing. (2019).

5 Wood, S. N. A simple test for random effects in regression models. *Biometrika* **100**, 1005-1010, doi:10.1093/biomet/ast038 (2013).

**Supplement 3 – Partial Constrained Principal Coordinate Analyses**

We used partial constrained principal coordinate analyses (hereafter PCoA) to relate data on bacterial taxa to experimental field treatments. Using partial constrained PCoA (a form of multivariate multiple regression that involves variance decomposition of unconstrained PCoA ^[^metric multidimensional dimensional scaling; MDS; ^16]^ we compared the taxonomic matrix to a matrix of experiment variables. Unlike MDS, in which orthogonal eigenvectors explain all variation in the taxonomic response matrix computed from some metric of species dissimilarities among sites, PCoA first removes variation due to specified random effects (conditioning variables), then calculates the portion of variation due to a linear combination of explanatory effects (constraining variables), with one eigenvector per degree of freedom; the remainder of variation is explained by the MDS ^[17]^. Each eigenvector is an axis in multidimensional space explaining some degree of variation, where the first axis explains the most variation, etc. Models were conducted in R v3.6 with package vegan v2.4 ^[14,18]^.

Our PCoA models explored the importance of experimental context and soil properties. We conditioned our analyses on spatial variation effects evaluated through both the in-field pairing of burnt/unburnt plots and soil type as described in Supplement 2. Categorical linear constraints included 2014 fire status (burnt/unburnt), substrate (litter/surface soil), pine proximity (near/away from overstory pines), and the interactive effects of these three experimental treatments. Continuous constraining effects included soil properties: phosphorus, nitrogen, organic carbon, and ground water content. The PCoA used Euclidian distances of the bacterial ESV response matrix. Prior to running the PCoA model, we linearized relative abundances of bacterial taxa that were already standardized to the geometric mean with a Hellinger transformation ^[19]^. Though normally used to down-weight less abundant taxa, the mean centered data already had potentially very low values logarithmically less than 1; the Hellinger transformation helped to restore more comparable relative abundances rather than further down-weighting.

We compared PCoA across taxonomic levels from genus to phylum to determine which provided the greatest resolution of treatment effects. We compared the output from each model, and we considered a level to be optimum if it maintained relationships of lower levels, had significant effects and at least first two axes, and had superior R^2^. We accounted for community correlations between paired plots and across space by conditioning on pairs and the soil type principal component, respectively. Models provided similar differentiation among treatments based on genus and family; burnt and unburnt treatments had increasingly higher overlap within litter and soil groups at progressively higher taxonomic levels. As measured by permutation F-tests against residual sums of squares, family-level PCoA linear constraint sums of squares were proportionately higher than at all higher taxonomic levels. Although fire effects were similar for family and genus level analyses, the substrate effect was 37% higher for family than genus. Class-adjusted R^2^ values were similar for family and genus (0.74 and 0.72, respectively), but Class-adjusted R^2^ values for all other levels were lower (~0.65). The only exception was for class, where the proportion explained by substrate was 11% higher than for family; however, the proportion explained by fire and the interaction between fire and substrate were much higher for family than for class (54% and 20% higher, respectively). Finally, the family model had the lowest proportion of variance unexplained by the combined linear combination and conditional effects. As a result, we focused on the PCoA that mapped plots based on site and taxonomic family scores. Site and family scores were determined using Euclidean distances, with family differences square root transformed.

We interpreted PCoA graphically as a 2-dimensional representation of multidimensional site-taxa and explanatory variable relationships. In PCoA, axes are both linear combinations of response variables and linear constraints. Categorical variables and interactions have foci at designated centroids, while linear variables are continuous across the axes. Each constrained axis is an eigenvector associated to some degree of variation with constraining variables. The magnitude of site or taxa values (scores) on each axis is a direct measure of association with variables explaining the variation of that axis; large scores denote strong associations with that part of the axis, and thus to associated variables. We visualized the first two axes through associating sites conditional on taxa, where the proximity of each site ordinate was centered among the taxa ordinates most associated with that site ^[sometimes referred to as scaling 1; 16,17]^.

We considered taxa that are strongly associated with categorical effects to be particularly influential to community differentiation. Following the ordination, we identified all influential taxa for further analysis via a generalized linear mixed model (GLMM) to explore more nuanced relationships and potentially greater variance explained than ordination alone provides ^[20]^. We defined influential taxa as any taxa with absolute value of scores greater than the mean of distance from the origin to interaction centroids. If a taxon ordinate was located beyond the bounds of a threshold of this distance, then the PCoA suggested that taxon was more strongly associated with experimental treatments than most other taxa. In effect, influential taxa are those associated with sites that have the most distinct communities relative to linear constraints. To facilitate interpretation of the ordination, we included this threshold as a circle in the ordination figure.

We used PCoA family scores to identify influential families. Influential families were families that had PCoA family scores on one of the two axes greater than two standard deviations above mean scores. Prior to model fit, the abundance for each family was divided by the mean abundance of that family such that the mean abundance of the model data is 1 for all families. Thus, interpretation of effects is the multiple of the mean from zero effect on the log scale. We determined differences of relative abundances, within each influential family, among treatments with a generalized linear mixed model (GLMM) that analyzed families simultaneously with a within-taxon standardization and allowed inferences of each family's relative abundance among treatments but not among other families. Due to the standardization, the families considered as part of this analysis occurred in all treatments at least once, not necessarily all plots. Influential families are thus certain families that showed strong differences in their relative abundance among treatments, which then substantially influenced plot-level ordination on the PCoA. This method is somewhat similar in principle to indicator species analyses used by others to identify taxa that were predominantly found in certain treatments ^[21,22]^, except that the use of influential families seeks comparisons of the relative abundances correlated to treatments of families found in all treatments. The model tested for differences within taxa, rather than among them, and all significance tests using simultaneous confidence intervals were adjusted only for the number of treatments included. Thus, the influential families compare relative abundances of a single family between different treatments. The GLMM fit the triple interaction among fire, substrate, and taxa to a Tweedie distribution, a mixture of Poisson and Gamma distributions that accounts for large numbers of zeroes in count or continuous data and that can model data that have many zeros, but are otherwise Poisson distributed ^[23]^. We used pack mgcv v1.8 in R 3.5 ^[14,24]^.

We explored relationships in overall abundance and frequency of occurrence between grouped influential and noninfluential families. We compared means of each metric and modelled percentile rank means regressed on relative occurrence. This allows us to consider the rank abundance of a family that was or was not determined to be influential, as well as that family’s frequency of occurrence across all plots regardless of treatment. We tested for differences in mean abundance and occurrence using Welch’s two sample t-tests to account for unbalanced sample sizes and marginally similar variances. Then we tested if there were differences in abundance conditional on occurrence between the taxa groups by modeling the relative odds ratios of percentile rank abundance regressed on occurrence using a beta GLM with logit link; because the highest percentile rank was 1, we first subtracted 0.001 from percentiles to fit the distribution support. We used base R for t-tests and package mgcv v1.8 for the beta regression in R v3.5 ^[14,24]^.

**Supplement 3 - References**

1 Peet, R. K., Platt, W. J. & Costanza, J. K. in *Ecology and Recovery of Eastern Old-Growth Forests* (eds Andrew M. Barton & William S. Keeton) 39-62 (Island Press/Center for Resource Economics, 2018).

2 Noel, J., Platt, W. J. & Moser, E. Structural Characteristics of Old‐ and Second‐Growth Stands of Longleaf Pine (Pinus palustris) in the Gulf Coastal Region of the U.S.A. *Conservation Biology* **12**, 533-548, doi:10.1111/j.1523-1739.1998.96124.x (1998).

3 Noss, R. F. *et al.* How global biodiversity hotspots may go unrecognized: lessons from the North American Coastal Plain. *Diversity and Distributions* **21**, 236-244, doi:<https://doi.org/10.1111/ddi.12278> (2015).

4 Lawton, D. E. M., W.E.; Moye, F.J.; Murray, J.B.; O’Connor, B.J.; Penley, H.M.; Sandrock, G.S.; & Friddell, M. S. H., J.H.; Huddlestun, P.F.; et al. (Georgia Department of Natural

Resources and Georgia Geological Survey, Atlanta, GA, USA, 1976).

5 Sanders, T. E. *Soil Survey of Leon County, Florida*. 151 (US Department of Agriculture, Soil Conservation Service and Forest Service, 1981).

6 Robertson, K. M., Platt, W. J. & Faires, C. E. Patchy Fires Promote Regeneration of Longleaf Pine (Pinus palustris Mill.) in Pine Savannas. *Forests* **10**, doi:ARTN 367

10.3390/f10050367 (2019).

7 Rother, M. T., Huffman, J. M., Guiterman, C. H., Robertson, K. M. & Jones, N. A history of recurrent, low-severity fire without fire exclusion in southeastern pine savannas, USA. *Forest Ecology and Management* **475**, 118406, doi:<https://doi.org/10.1016/j.foreco.2020.118406> (2020).

8 Platt, W. J., Evans, G. W. & Rathbun, S. L. The Population Dynamics of a Long-Lived Conifer (Pinus palustris). *The American Naturalist* **131**, 491-525 (1988).

9 Platt, W. J. & Rathbun, S. L.

10 Mugnani, M. P., Robertson, K. M., Miller, D. L. & Platt, W. J. Longleaf Pine Patch Dynamics Influence Ground-Layer Vegetation in Old-Growth Pine Savanna. *Forests* **10**, 389 (2019).

11 Bouyoucos, G. J. Hydrometer Method Improved for Making Particle Size Analyses of Soils. *Agronomy Journal* **54**, 464-465, doi:<https://doi.org/10.2134/agronj1962.00021962005400050028x> (1962).

12 Wood, S. N. Fast stable restricted maximum likelihood and marginal likelihood estimation of semiparametric generalized linear models. *Journal of the Royal Statistical Society: Series B (Statistical Methodology)* **73**, 3-36, doi:<https://doi.org/10.1111/j.1467-9868.2010.00749.x> (2011).

13 Wood, S. N., Pya, N. & Säfken, B. Smoothing Parameter and Model Selection for General Smooth Models. *Journal of the American Statistical Association* **111**, 1548-1563, doi:10.1080/01621459.2016.1180986 (2016).

14 Team, R. C. R: A language and environment for statistical computing. (2019).

15 Wood, S. N. A simple test for random effects in regression models. *Biometrika* **100**, 1005-1010, doi:10.1093/biomet/ast038 (2013).

16 Legendre, P. & Legendre, L. *Numerical ecology*. (Elsevier, 2012).

17 Zuur, A., Ieno, E. N. & Smith, G. M. *Analyzing ecological data*. Vol. 75 (Springer, 2007).

18 Oksanen, J. *et al.* Vegan: Community Ecology Package. *R Package Version 2.2-1* **2**, 1-2 (2015).

19 Legendre, P. & Gallagher, E. D. Ecologically meaningful transformations for ordination of species data. *Oecologia* **129**, 271-280, doi:10.1007/s004420100716 (2001).

20 Borcard, D., Gillet, F. & Legendre, P. *Numerical ecology with R*. (Springer, 2018).

21 Barreto, C. R., Morrissey, E. M., Wykoff, D. D. & Chapman, S. K. Co-occurring Mangroves and Salt Marshes Differ in Microbial Community Composition. *Wetlands* **38**, 497-508, doi:10.1007/s13157-018-0994-9 (2018).

22 Huffman, M. S. & Madritch, M. D. Soil microbial response following wildfires in thermic oak-pine forests. *Biology and Fertility of Soils* **54**, 985-997 (2018).

23 Jorgensen, B. Exponential Dispersion Models. *Journal of the Royal Statistical Society. Series B (Methodological)* **49**, 127-162 (1987).

24 Wood, S. Package ‘mgcv’. *R package version* **1**, 29 (2015).

**Supplement 4 – Family Choice**

Methods were identical across taxonomic levels. Each PCoA used the same standardizations and equations (Supplementary Fig. S3). Taxonomy through domain was assigned to all ESVs used for all levels (described in Methods).

Family is more ecologically interpretable than other taxonomic levels. Treatment differences are similar between genera and families, but as levels increase, taxa become more homogeneous among substrate and fire treatments. This is due to within-group correlations, made clear by analyzing influential families (Supplementary Fig. S3D).

Family-level relationships preserve genus-level relationships among treatments that are lost in higher orders, while high dispersion in higher order taxonomic groups negates robust inferences at these levels. Genus taxon-plot relationships are somewhat underdispersed, as seen by the large number of taxa that do not contribute to distances due to low relative occurrence, therefore making inferences among genera difficult. However, among families frequencies are high enough that inferences become much more robust (Supplementary Fig. S3D). As levels increase, apparent taxon relationships to plots become overdispersed. The standard deviations of taxa distances from the origin increase as levels increase (Supplementary Fig. S3; 2SD black circles) with corresponding spread of taxonomic distances (Supplementary Fig. S3; blue squares). Consequently, differences among plots and, therefore, treatments become smaller. Although overall community effects are strong among genera, inferences among specific taxa are difficult and suspect. As levels increase, taxon relationships to plots become highly treatment-specific, while paradoxically creating substantial overdispersion as by increasingly large 95% intervals that extend well beyond treatment centroids. This paradox is driven by presence or absence of phylogenetic correlation.

Effects are artificially lost in the absence of phylogenetic correlation and inflated in the presence. As taxonomic levels increase, differences among effects shrink. With each successive levels, plots overall, regardless of treatment, become more similar to others (Supplementary Fig. S3; closer to origin). At the same time, 95% intervals increase dramatically and extend well beyond treatment centroids (Supplementary Fig. S3; large circles). The fire-substrate interaction is lost as burned and unburned litter, in particular, lose identifiability (Supplementary Fig. S3A-E; overall decrease in axis 2 variance explained). Soils homogenize to a lesser, but still noticeable, extent. Identifiability loss occurs where families within a higher taxon independently lean towards or strongly favor a single fire-substrate effect (lack phylogenetic correlation; i.e., families within class Alphaproteobacteria), and effects get subsumed into the overall abundance of the respective higher order level (e.g., for orders Frankiales and Rhizobiales, class Gammaproteobacteria, and phylum Actinobacteria). Dispersion becomes driven by the least common taxa (e.g., Archaea and phylum Verrucomicrobia), thereby losing most information in lower levels.

The choice of family level relationships facilitates greater interpretability of the GLMM. While separations by substrate and fire, with an interaction between them, are evident at the genus level, the response matrix is sparse due to high numbers of zeros in abundances, with few taxa contributing to the dissimilarity matrix. For this reason, genera overall provide little information on influential taxa as seen by high numbers of taxa between treatment clusters with few apparent contributions by individual genera. This indicates underdispersion. Families contain much more robust numbers of positive abundances than genera. Thus, we trust dissimilarities more than genera-derived indices as among-family contribution is stronger. Influential taxa are much clearer. At the same time, ecological differences are preserved, suggesting that this level contains similar information as genus but without underdispersion.

It is clear from the GLMM analyzing family associations with fire and substrate that for orders with multiple families there are differences in family effects. These associations become lost when examining organization at order level, with more abundant families dominating apparent but erroneous effects. As taxonomic levels increase, this correlation becomes more pronounced. Overdispersion is apparent as taxonomic associations with treatment become singular, with taxon points beyond treatment centroids. Additionally, this correlation results in loss of information with higher taxonomic levels as distances between centroids become smaller and the fire-substrate interaction disappears. This can be seen by comparing distances to the 2SD family circle.

# **Figure Legends**

**Supplementary Figure S1**. **Wade Tract and Field Experiment Layout.** Left: Location of the Wade Tract Preserve in Thomas County, southwest Georgia, USA. Right: Distribution of experimental plots within two burn units comprising the 85 ha old-growth section of the conservation easement. Following 2014 prescribed fires, 30 unburnt plots (U) and paired burnt plots (B) were selected in upland regions of the easement. Paired plots were randomly selected based on proximity to overstory longleaf pine: 15 paired plots >10 m from pines (yellow) and 15 paired plots <5 m from pines (blue). Aerial map of Wade Tract courtesy of Neil Jones.

**Supplementary Figure S2. Soil properties in Wade Tract plots.** For each variable (A-F above graphs: 1) the left graph shows the mean values of the measured property (with 95% CI) at each level of fire and pine treatments (U: unburnt plots; B: burnt plots; A: plots >5m from overstory pines; N: plots <5m from overstory pines; *n*=15); 2) the right graph shows the mean paired ratio U:B values (with 95% CI), where for each variable the value 1 (dotted line) indicates similar values of the variables in the unburnt and burnt plots. Data are from Tables S2-1-S2-4.

**Supplementary Figure S3. Partially constrained principal coordinates analyses (PCoA) showing dispersion of taxa from genus to phylum level.** Circles are 95% standard deviation limits among taxon distances. Graphs are based on the first two constrained axes of PCoA. Percent of total constrained variation explained by each axis is given in parentheses. Triangles - litter samples; circles-adjacent soil samples. Red - burnt samples; blue - unburnt samples. Small cyan squares indicate individual taxa.

**Supplementary Table S1.** **Overall *Fire* effect contrasts by chemistry for Z-transformed soil values.** Changes between treatments are back transformed to measurement scale, where ratios and differences are mean unburnt values divided by and minus mean burnt values, respectively. Water content, nitrogen (%), and organic carbon (%) are 1-tailed tests that burnt plots have lower values than unburnt; the others are 2-tailed tests that effects differ between fire treatments. Tests are Wald contrasts with $\chi_{df=1}^{2}$. Asterisks denote significance at different levels of P-value that are Bonferroni adjusted within chemistry for second test with pine proximity contrasts and for respective tailed tests; tests are orthogonal among chemistries so are not further adjusted (^*^ = 0.1; ^**^ = 0.05; ^***^ = 0.001).

**Supplementary Table S2.** **Overall *Pine proximity* effect contrasts by chemistry for model fit to Z-transformed soil values**. Changes between treatments are back transformed to measurement scale, where ratios and differences are mean unburnt values divided by and minus mean burnt values, respectively. All tests are 2-tailed tests. Tests are Wald contrasts with $\chi_{df=1}^{2}$. Asterisks denote significance at different levels of P-value that are Bonferroni adjusted within chemistry for second test with fire contrasts and for respective tailed tests; tests are orthogonal among chemistries so are not further adjusted (^*^ = 0.1; ^**^ = 0.05; ^***^ = 0.001).

**Supplementary Table S3.** **Relationships of overall mean values for each soil chemistry for *Fire + Pine proximity* effects, combined from tables S2-1 and S2-2 for clarity.** Changes between treatments are back transformed to measurement scale, where ratios and differences are average unburnt values divided by and minus mean burnt values, respectively. 95% confidence intervals for treatment combinations are given in parentheses. Asterisks denote significance of fire effect contrasts (Table S2).

**Supplementary Table S4.** **Tests that ratios of unburnt/burnt plots and away/near pines plots differ from one.** Ratios are estimated using the geometric mean unburnt value for each respective group to account for skewed distributions and potential differences in unburnt values between pine treatments. Ratios (U/B): water content, nitrogen, and organic carbon are 1-tailed tests that burnt plots are proportionately lower than unburnt (ratio > 1), the others are 2-tailed tests that ratios differ from 1. Ratios (A/N): all tests are 2-tailed that pine proximity ratios differ. Tests are Wald $\chi_{df=1}^{2}$. Asterisks denote significance at different levels of P-value with Bonferroni adjustments for three tests, 2 within pine proximity and one between. Significance indicated by asterisks account for multiple and respective tailed tests; tests are orthogonal among chemistries so are not further adjusted (^*^ = 0.1; ^**^ = 0.05; ^***^ = 0.001).

# **Supplementary Tables**

**Supplementary Table S1.** **Overall Fire effect contrasts by chemistry for Z-transformed soil values.**

|  | **Contrast** | | | |  | **Fire effect** | |  |  | |
| --- | --- | --- | --- | --- | --- | --- | --- | --- | --- | --- |
| **Chemistry** | **Unburnt** | | **Burnt** | | | **Ratio (U/B)** | **Difference (U-B)** | $\boldsymbol{\chi}_{\boldsymbol{1}}^{\boldsymbol{2}}$ | **P-value** | |
| pH | 6.28 |  | 6.27 |  | | 1.00 | 0.02 | 0.48 | 0.4882 |  |
| Water content (%) | 23.79 |  | 17.92 |  | | 1.33 | 5.87 | 9.05 | 0.0026 | ^***^ |
| Phosphorus (ppm) | 23.32 |  | 23.17 |  | | 1.01 | 0.15 | 0.00 | 0.9765 |  |
| Nitrogen (%) | 0.15 |  | 0.12 |  | | 1.25 | 0.03 | 10.55 | 0.0012 | ^***^ |
| Organic carbon (%) | 9.95 |  | 8.46 |  | | 1.18 | 1.48 | 4.14 | 0.0419 | ^**^ |
| Carbon:Nitrogen | 60.6 |  | 66.28 |  | | 0.91 | -5.68 | 3.14 | 0.0763 |  |

**Supplementary Table S2.** **Overall Pine proximity effect contrasts by chemistry for model fit to Z-transformed soil values.**

|  | **Contrast** | | | |  | **Pine effect** | |  |  | |
| --- | --- | --- | --- | --- | --- | --- | --- | --- | --- | --- |
| **Chemistry** | **Away** | | **Near** | | | **Ratio (A/N)** | **Difference (A-N)** | $\boldsymbol{\chi}_{\boldsymbol{1}}^{\boldsymbol{2}}$ | **P-value** | |
| pH | 6.29 |  | 6.27 |  | | 1.00 | 0.02 | 0.68 | 0.408 |  |
| Water content (%) | 22.37 |  | 19.35 |  | | 1.16 | 3.02 | 2.39 | 0.1219 |  |
| Phosphorus (ppm) | 29.17 |  | 17.32 |  | | 1.68 | 11.85 | 5.44 | 0.0196 | ^**^ |
| Nitrogen (%) | 0.15 |  | 0.13 |  | | 1.09 | 0.01 | 1.73 | 0.189 |  |
| Organic carbon (%) | 8.76 |  | 9.65 |  | | 0.91 | -0.9 | 1.51 | 0.2184 |  |
| Carbon:Nitrogen | 59.79 |  | 67.08 |  | | 0.89 | -7.29 | 5.18 | 0.0229 | ^**^ |

**Supplementary Table S3.** **Relationships of overall mean values for each soil chemistry for Fire + Pine proximity effects, combined from Supplementary Tables 1 and 2 for clarity.**

|  | | | | | | | | |
| --- | --- | --- | --- | --- | --- | --- | --- | --- |
| **Chemistry** | **Pine** | **Unburnt** | | **Burnt** | | **Difference (U-B)** | **Ratio (U/B)** | |
| pH | Away | 6.29 | (6.25, 6.34) | 6.28 | (6.24, 6.32) | 0.02 | 1.00 |  |
|  | Near | 6.27 | (6.23, 6.32) | 6.26 | (6.22, 6.3) |  |  |  |
|  |  |  |  |  |  |  |  |  |
| Water content (%) | Away | 25.3 | (21.98, 28.62) | 19.43 | (16.21, 22.65) | 5.87 | 1.33 |  |
|  | Near | 22.28 | (18.95, 25.62) | 16.42 | (13.12, 19.72) |  |  |  |
|  |  |  |  |  |  |  |  |  |
| Phosphorus (ppm) | Away | 29.24 | (20.78, 37.71) | 29.09 | (20.89, 37.3) | 0.15 | 1.01 |  |
|  | Near | 17.39 | (8.57, 26.22) | 17.25 | (8.46, 26.03) |  |  |  |
|  |  |  |  |  |  |  |  |  |
| Nitrogen (%) | Away | 0.16 | (0.14, 0.18) | 0.13 | (0.11, 0.15) | 0.03 | 1.25 |  |
|  | Near | 0.15 | (0.13, 0.16) | 0.12 | (0.1, 0.13) |  |  |  |
|  |  |  |  |  |  |  |  |  |
| Organic carbon (%) | Away | 9.5 | (8.26, 10.74) | 8.01 | (6.81, 9.22) | 1.48 | 1.18 |  |
|  | Near | 10.39 | (9.15, 11.64) | 8.91 | (7.68, 10.14) |  |  |  |
|  |  |  |  |  |  |  |  |  |
| Carbon:Nitrogen | Away | 56.95 | (51.5, 62.41) | 62.64 | (57.35, 67.92) | -5.68 | 0.91 |  |
|  | Near | 64.24 | (58.76, 69.72) | 69.92 | (64.5, 75.34) |  |  |  |

**Supplementary Table S4. Tests that ratios of unburnt/burnt plots and away/near pines plots differ from one.**

|  |  | **Ratio (Unburnt/Burnt)** ≠ 1 | | | | | |  | **Ratio (Away/Near)** ≠ 1 | | | | | |
| --- | --- | --- | --- | --- | --- | --- | --- | --- | --- | --- | --- | --- | --- | --- |
| **Chemistry** | **Pine** | **Ratio** | **(LCL, UCL)** | | $\boldsymbol{\chi}_{\boldsymbol{1}}^{\boldsymbol{2}}$ | **P value** | |  | **Ratio** | **(LCL, UCL)** | $\boldsymbol{\chi}_{\boldsymbol{1}}^{\boldsymbol{2}}$ | **P value** | |  |
| pH | Away | 1.02 | | (0.91, 1.14) | 0.12 | 0.7273 |  |  | 1.01 | (0.87, 1.18) | 0.02 | 0.9005 |  |  |
|  | Near | 1.01 | | (0.91, 1.12) | 0.04 | 0.8420 |  |  |  |  |  |  |  |  |
|  |  |  | |  |  |  |  |  |  |  |  |  |  |  |
| Water content | Away | 1.17 | | (1.05, 1.30) | 8.39 | 0.0038 | *** |  | 1.00 | (0.86, 1.16) | 0.00 | 1.0000 |  |  |
|  | Near | 1.17 | | (1.05, 1.29) | 8.58 | 0.0034 | *** |  |  |  |  |  |  |  |
|  |  |  | |  |  |  |  |  |  |  |  |  |  |  |
| Phosphorus | Away | 0.97 | | (0.87, 1.08) | 0.33 | 0.5644 |  |  | 1.26 | (1.08, 1.47) | 8.79 | 0.0030 | *** |  |
|  | Near | 0.77 | | (0.69, 0.87) | 18.93 | <0.0001 | *** |  |  |  |  |  |  |  |
|  |  |  | |  |  |  |  |  |  |  |  |  |  |  |
| Nitrogen | Away | 1.27 | | (1.14, 1.41) | 19.72 | <0.0001 | *** |  | 1.11 | (0.96, 1.29) | 2.07 | 0.1505 |  |  |
|  | Near | 1.14 | | (1.03, 1.26) | 6.06 | 0.0138 | ** |  |  |  |  |  |  |  |
|  |  |  | |  |  |  |  |  |  |  |  |  |  |  |
| Organic carbon | Away | 1.27 | | (1.14, 1.41) | 19.83 | <0.0001 | *** |  | 1.20 | (1.04, 1.39) | 5.94 | 0.0148 | ** |  |
|  | Near | 1.06 | | (0.95, 1.17) | 1.06 | 0.3040 |  |  |  |  |  |  |  |  |
|  |  |  | |  |  |  |  |  |  |  |  |  |  |  |
| Carbon:Nitrogen | Away | 0.99 | | (0.90, 1.10) | 0.01 | 0.9198 |  |  | 1.08 | (0.92, 1.25) | 0.89 | 0.3464 |  |  |
|  | Near | 0.92 | | (0.83, 1.02) | 2.80 | 0.0941 |  |  |  |  |  |  |  |  |
