# Supplementary figures and images for: Substrate and low intensity fires influence bacterial communities in longleaf pine savanna
Source: Sci Rep. 2022 Dec 3;12:20904. doi: 10.1038/s41598-022-24896-x (PMC9719495; doi:10.1038/s41598-022-24896-x)

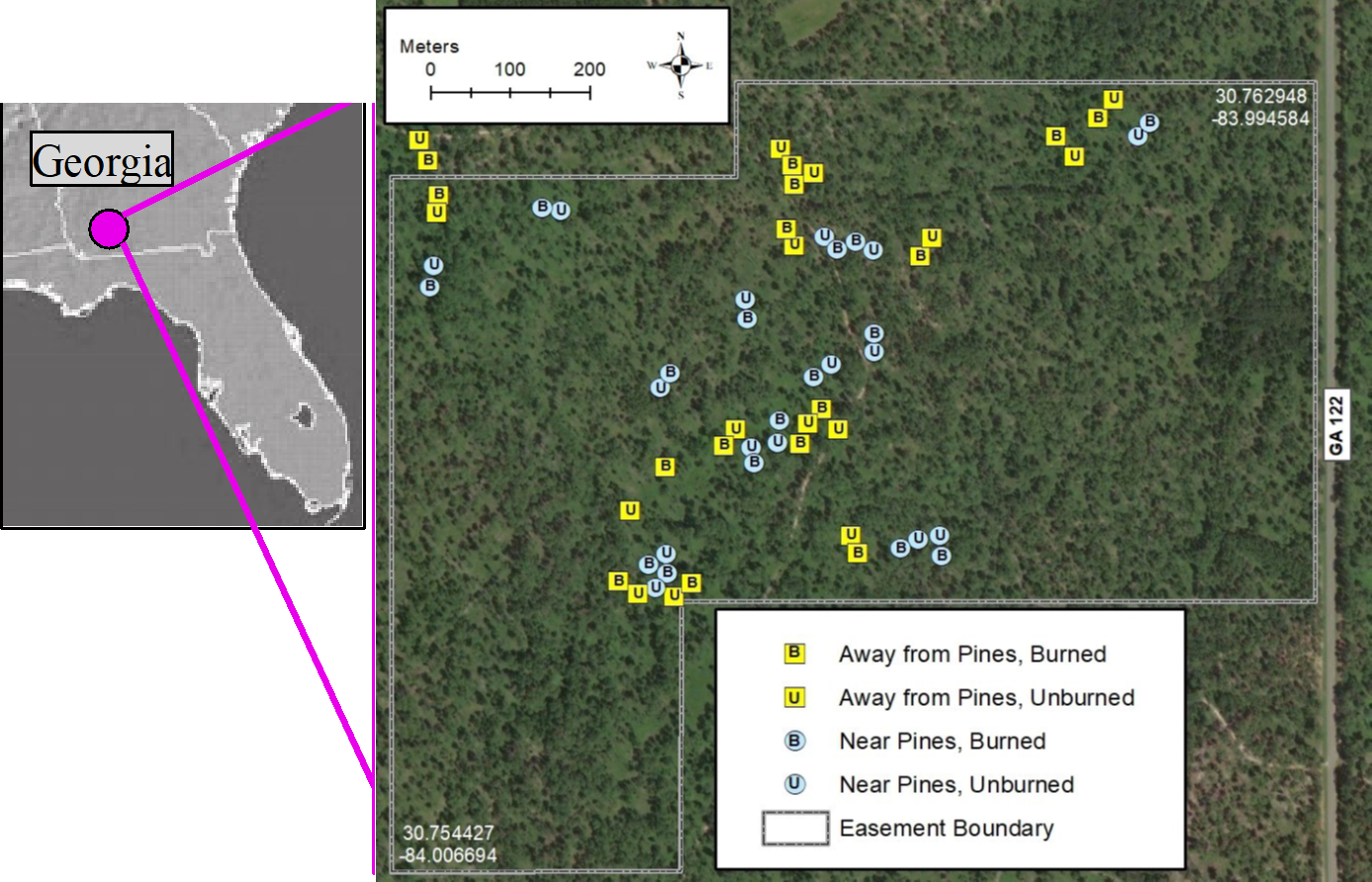

Supplement: Supplementary file 2 — Supplementary Figure S1. [file 41598_2022_24896_MOESM2_ESM.tif]

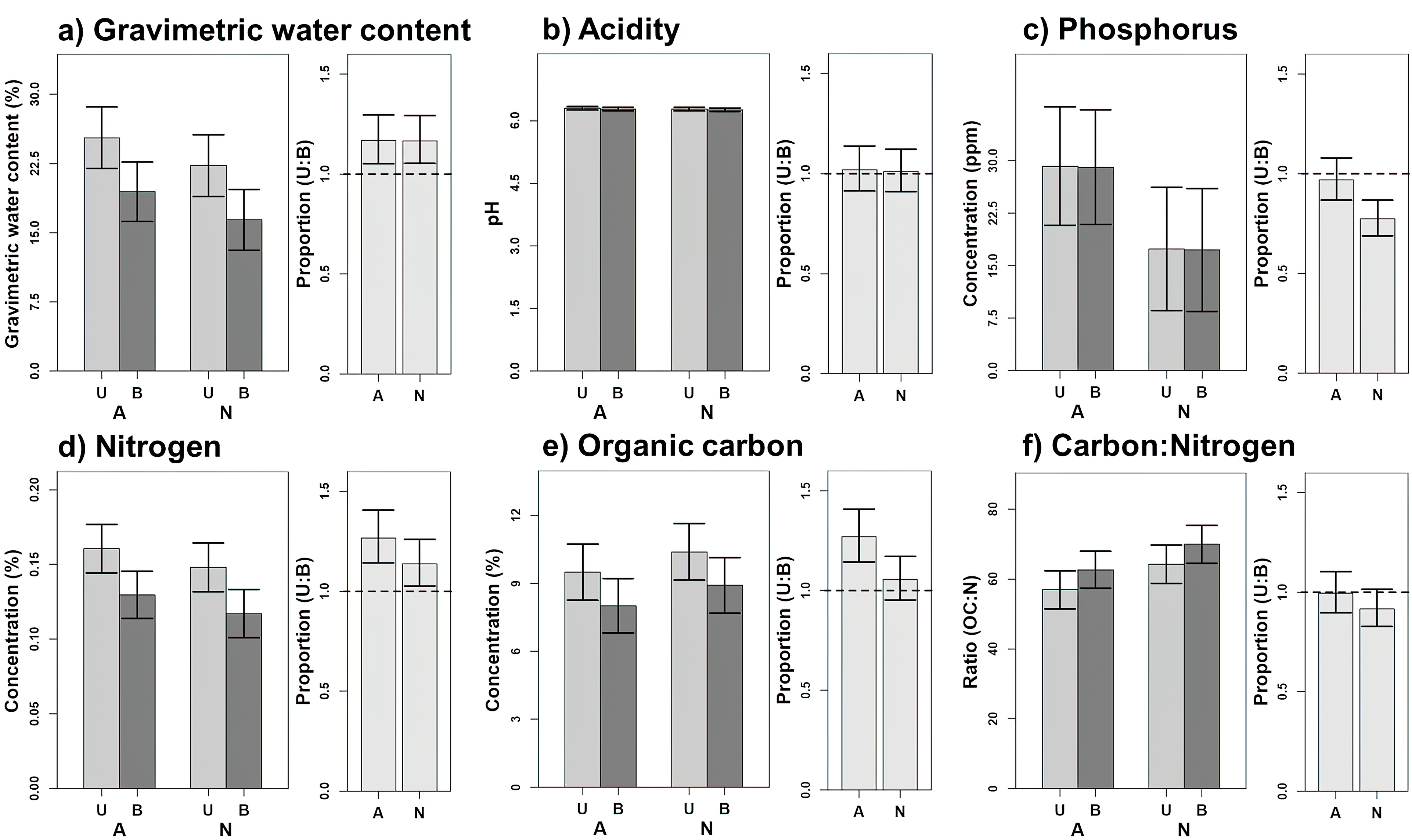

Supplement: Supplementary file 3 — Supplementary Figure S2. [file 41598_2022_24896_MOESM3_ESM.tif]

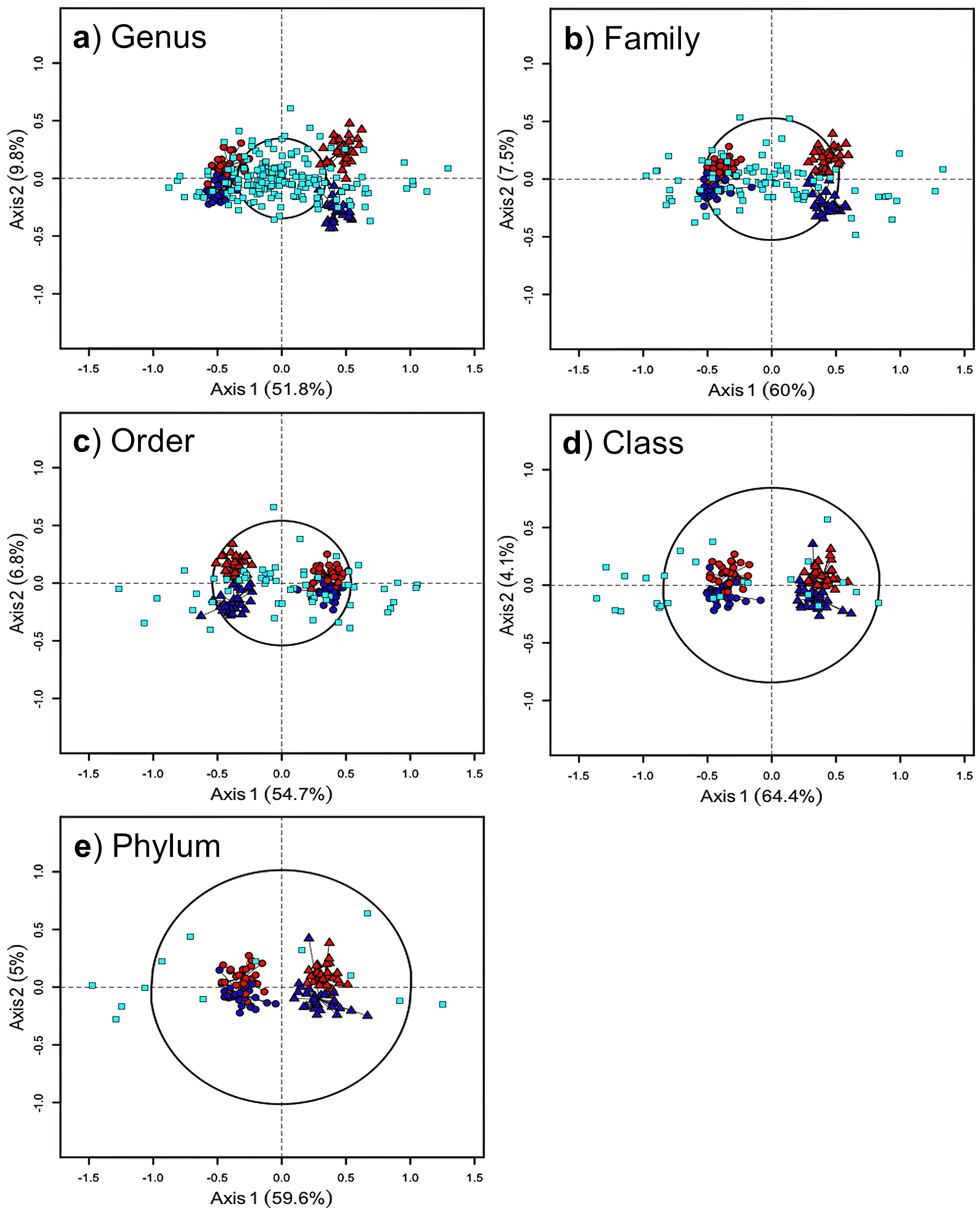

Supplement: Supplementary file 4 — Supplementary Figure S3. [file 41598_2022_24896_MOESM4_ESM.tif]
